# Source node management in complex water network based on cascading failure and bidirectional risk transmission: The case of Henan Province

**DOI:** 10.1371/journal.pone.0358079

**Published:** 2026-10-08

**Authors:** Junhua Zhang, Ran Jing, Jing Dai, Yaohong Yang, Runpeng Wei, Junyan Gao

**Affiliations:** 1 School of Surveying and Geo-Informatics, North China University of Water Resources and Electric Power, Zhengzhou, China; 2 School of Water Conservancy, North China University of Water Resources and Electric Power, Zhengzhou, China; 3 School of Management and Economics, North China University of Water Resources and Electric Power, Zhengzhou, China; Nanjing University of Science and Technology, CHINA

## Abstract

Water source nodes within water network are subject to dual pressures: the propagation of failure risks and reverse feedback from fluctuations in user demand. As the connectivity of water network and the relationship between water supply and consumption become increasingly complex, there is an urgent need to elucidate the patterns of bidirectional risk transmission in complex water network and to effectively manage water source nodes. Taking the water network of Henan Province as a case study, improved forward and reverse cascade failure models were established respectively. The criticality of water source nodes in forward risk propagation and their sensitivity to pressure in reverse feedback were comprehensively identified. Spearman correlation analysis and K-means clustering were employed to classify water source nodes into ‘criticality+sensitivity’ two-dimensional clusters. The results indicate that forward risk transmission exhibits distinct head-concentration characteristics, with a small number of critical reservoir nodes dominating cascade failure. Spatially, risk first propagates through the structural layer before permeating to the functional endpoints.Temporally, it follows a non-linear evolution pattern, initially intensifying and subsequently attenuating. Reverse risk propagation exhibits characteristics of ‘source-proximity priority’ and hierarchical feedback: as the degree of demand deviation and the number of affected users increase, reservoirs closest to the source are the first to reach their pressure limits and transmit pressure to secondary reservoirs. The two-dimensional classification criteria combining the criticality and sensitivity of reservoir nodes more comprehensively reflect the characteristics of reservoir nodes within the water network, enabling more precise, differentiated management of these nodes. This study broadens the research perspective on risk propagation in complex water network and provides theoretical support and decision-making guidance for the precise management of water source nodes to enhance the water supply security of water network.

## Introduction

With the successive completion and commissioning of cross-regional and cross-basin water transfer projects such as the South-to-North Water Diversion Project, the Yangtze River to Huai River Water Diversion Project, and the Han River to Wei River Water Diversion Project, the main framework of China’s national water network is gradually taking shape. The pattern of water resource allocation continues to be optimised, and the overall capacity to ensure water security has been significantly enhanced [1]. Located in central-eastern China, Henan Province is a vital region connecting the east, centre and west of the country and linking the north and south. It is also a key area for implementing national strategies such as ecological protection and high-quality development in the Yellow River Basin, as well as the high-quality development of follow-up projects for the South-to-North Water Diversion Project. Consequently, water supply security in Henan Province is of major strategic significance, as it not only concerns regional socio-economic stability but also directly impacts national food security, the stability of key industrial chains, and the security of cross-regional water resource allocation. Henan Province spans the four major river basins of the Yellow River, the Huai River, the Hai River and the Yangtze River. The natural water systems of these four basins, together with artificial water diversion projects such as the Central Route of the South-to-North Water Diversion Project and the Yellow River Diversion Project, as well as regional reservoirs and urban and rural water supply networks, collectively form a complex water network. Numerous practical factors, including increasingly severe climate change, frequent extreme hydrological events, rapid urbanisation and the growing connectivity of water systems, have increased the probability of risks occurring within the water network system and amplified the severity of their consequences. When local critical nodes are affected by extreme events or operational disturbances, risks are transmitted and propagated throughout the network via the interconnections within the water system. The heterogeneous nature of water network means that risk propagation exhibits complex characteristics, such as amplification at critical nodes and local failures triggering large scale impacts. Therefore, analyzing the patterns of risk propagation in water network to provide a scientific basis for their management holds significant theoretical and practical value.

Risks exhibit bidirectional propagation within water network. They can propagate from the water source to the water user end along the connections within the water system, and they can also propagate in the opposite direction.Accordingly, risks within water network systems are categorized into two main types: forward transmitted risks and reverse-transmitted risks. Forward-transmitted risks include: water quantity risks such as insufficient water volume, interruptions in water supply and reduced water supply capacity; water quality risks triggered by sudden point source pollution; the downstream accumulation of pollutants and the resuspension of pollutants from bottom sediments; flood risks caused by the downstream discharge of flood peaks and reservoir overfilling; and operational risks arising from failures in reservoirs, sluice gates, pumping stations and water conveyance channels. Reverse transmission risks primarily manifest as supply pressures on upstream water source nodes caused by fluctuations in water demand. Such significant fluctuations in water demand stem from various factors: the concentration of population inflows into cities or high temperatures leading to increased domestic water use for bathing, cleaning and cooling; crops entering critical water-demand periods, with high temperatures and droughts causing increased irrigation water use; the expansion of urban industrial capacity, the growth of water-intensive industries, and the addition of new water intensive enterprises in industrial parks. Conversely, increased rainfall leading to a reduction in the three categories of water use, can all cause significant fluctuations in demand at the user end. Here, this impact arising from demand side disturbances and transmitted to the water source side is characterized as pressure feedback [2]. Given that water supply reliability risks may stem either from impaired supply capacity at the water source end or from fluctuations in demand at the user end, and that this approach effectively links the two processes of forward propagation and reverse feedback, we have selected differential water volume risk to characterize both water supply capacity failure and fluctuations in user demand, thereby analyzing the bidirectional propagation patterns of risks within the water network.

When a risk event occurs at a source side node, the risk load released by that node propagates downstream along the water flow, through network connections and the redistribution of risk loads, this results in continuous propagation and amplification. The cascade failure model is capable of characterizing the dynamic evolution of the network following a local failure [3]. Consequently, it is reasonable to introduce the cascade failure model to simulate and analyse the patterns of risk propagation. However, when risk source nodes transform into water use end nodes and the direction of risk transmission changes, the network edges become pressure edges reflecting pressure relationships, and the attributes of the edges change. Therefore it is necessary to redefine the network edges based on the water network topology to generate a new pressure feedback network. In this pressure feedback network, pressure is also transmitted via network connections, and the cascade failure model remains applicable.

Accordingly, this paper constructs a directed weighted water network topological model using the Henan Province water network as an example, and analyses the patterns of forward risk propagation based on simulation results from the cascade failure model. Furthermore, based on the pressure feedback network, the patterns of reverse risk propagation are analyzed using results from a modified reverse cascade failure model. Given that the heterogeneity of water network may cause the same reservoir node to play different roles in the two processes, this study further establishes key indicators for forward risk propagation and reverse pressure sensitivity for reservoir nodes. Spearman correlation analysis is employed to identify the relationship between the two sets of results, and K-means clustering is used to classify reservoir nodes into six categories based on the dual dimensions of ‘criticality’ and ‘sensitivity’. On this basis, differentiated management strategies for water source nodes are proposed.

The main contributions of this study are threefold. First, from a supply—demand perspective, the nodes are classified into three categories—reservoir, confluence, and user nodes—and a directed and weighted complex water network is constructed by simultaneously incorporating the structural and functional attributes of nodes as well as edge weights. Second, the risk assessment of water-source nodes incorporates the reverse pressure induced by fluctuations in user demand, thereby extending the conventional one-dimensional assessment of forward node criticality into a two-dimensional criticality—sensitivity framework. Third, reservoir nodes are divided into different management zones based on the two indicators of criticality and sensitivity, and differentiated management strategies are proposed for each zone.

## Literature review

Existing research on water network risks has covered numerous dimensions, including economic, social, managerial, engineering {Guo Yongyong, 2022 #80} and ecological. Research on risk propagation has gradually expanded from the analysis of a single risk in a single project to the study of risk propagation involving the coupling of multiple risks. Focusing on a single risk within a specific project, risk research in the field of water resources systems has primarily centred on the management of man-made engineering structures [4–6] and risk assessment in the field of ecological risks [7,8]. Research subjects have largely focused on key water projects such as the South-to-North Water Diversion Project [9] and the Three Gorges Reservoir [10,11]. Research has expanded from single risk studies to coupled analyses of multiple risk types. Addressing the issue of numerous risk sources and multi-factor coupling leading to disasters in high slope construction, Jiang et al. utilized systems theory to analyse the coupled mechanisms and conduct risk management research involving multiple risks namely, human, physical, environmental and management factors [12]. Focusing on the catchment scale [13], Feng et al. constructed a multi-linkage network of risks in complex river basin water resource systems based on complex network theory, investigated the propagation pathways of different interrelated elements within the network, and, on this basis, developed a risk propagation model, designed risk propagation indicators and risk management strategies [14]. Existing research lacks studies on risk propagation from the perspective of water network systems, and there is an even greater lack of research into the patterns of risk propagation from a reverse perspective.

Taking into account the differences in water network types, distinct topological network structure models have been constructed: one category comprises inter-basin water network [15], and Fan et al., which, based on complex network theory, generalize water network into a ‘spine-branch-node’ topological structure, where the spine and branches correspond to river systems and water diversion projects, the nodes correspond to water storage and regulation projects and key cross sections [16]. One category comprises regional water network: Zhang et al. constructed undirected topological networks (with reservoirs and lakes as nodes, and rivers and canals as edges) [17]. Li et al. abstracted the nodes (water conservancy hubs) of the water network as nodes and the ‘backbone-branch’ (water conveyance channels) as edges to construct a topological network [18]. Existing studies that use complex network models to construct topological network structures for water network have only analyzed the resilience and vulnerability of the network under specific risk scenarios, namely when certain network characteristic indicators are compromised [19]. Furthermore, existing water network models do not take into account the attributes of nodes and edges within the topological network, resulting in a certain gap of discrepancy between the constructed topological network and the actual network.

Research into the patterns of risk propagation has established a comprehensive logic of ‘risk source identification analysis of propagation mechanisms assessment of propagation effects’ across multiple fields, including energy systems [20,21], ecosystems [22–25], transport networks [26–28], and shipping networks [29]. In the field of energy systems, Fu et al. took China’s carbon neutral energy transition system as their subject of study, dividing systemic risk into four heterogeneous subsystems economic, social, natural and digital and utilized a system dynamics model to simulate the risk propagation process [20]. Taking cascading effects into account, Liu et al., against the backdrop of the Ukraine crisis, constructed a two stage model of oil supply risk propagation and recovery to analyse the cascading effects of risk within the global oil trade network. Their research overcame the limitations of risk analysis based solely on bilateral trade between individual countries and revealed the role of indirect risk transmission via non-direct partners within energy trade networks [21]. In the field of ecosystems, Deng et al. focused on three types of soil ecosystems in China agricultural, natural and urban and quantified the cascading effects of microplastic risk transmission at a national scale [24]. Jing et al. focused on drought risk in the Xilingol temperate grasslands of Inner Mongolia, revealing a three tiered cascading mechanism of risk transmission involving meteorological drought, soil drought and eco-hydrological drought [25]. In the field of transport networks, Hu et al. constructed a theoretical framework and simulation model for risk transmission cascade effects from the perspective of complex system process safety. They revealed the chain reaction transmission pattern of ‘initial accident secondary disaster’ in LNG-powered vessels, clarifying the mechanisms by which multi-factor coupling drives cascades and identifying key control nodes [28]. In the field of shipping networks, Xu et al. combined the Motter-Lai load capacity model with a dynamic shipping traffic reallocation mechanism to investigate the cascading propagation of risk within the global container shipping network following the disruption of a single port. The widespread application of theories such as cascading failure [26,30], complex networks [31,32], and infectious disease models [33] The widespread application of these theories in research on risk propagation provides methodological insights for studies on risk transmission in water network.

Traditional research has largely assessed the importance of water source nodes based on unidirectional water supply processes, making it difficult to answer two key questions: firstly, what is the extent and severity of the cascading effects on the water network following risks at different reservoir nodes. Secondly, how does the sensitivity to pressure vary across different reservoir nodes in response to feedback from fluctuations in downstream demand. Consequently, determining how to comprehensively assess the forward risk propagation capacity and reverse pressure resilience of reservoir nodes, identify their characteristics and implement zoned management, is an urgent issue in the risk management of water source nodes within complex water network.The contributions of this study are as follows: First, it provides a new approach for constructing a more realistic water network model by considering the structural roles and functional differences of different types of nodes in risk transmission, characterizing betweenness centrality as a structural attribute of nodes, characterizing reservoir storage capacity and designed water supply scale at the user end as functional attributes, setting edge weights according to the resistance to risk propagation in different water conveyance channels. Second, it incorporates the reverse feedback of demand pressure from the water use end to the water source end into water network risk transmission analysis, and constructs indicators of forward transmission criticality and reverse pressure-bearing sensitivity to reveal bidirectional risk transmission patterns from the dimensions of risk output and pressure bearing. Third, it conducts a two-dimensional comprehensive evaluation of reservoir nodes to more accurately identify the characteristics of different reservoir nodes and achieve refined classification, thereby providing a decision-making basis for differentiated control and management of reservoir nodes, determination of management priorities, and allocation of risk prevention and control resources.

## Methodology

Based on the complex network characteristics of water network and the bidirectional transmission of water network risks, this paper constructs a water network model and employs a cascade failure model to analyse the patterns of bidirectional risk transmission. For the forward analysis, a directed weighted water network topological model was constructed. An improved forward cascading failure model is used to analyse the patterns of forward risk transmission. Reverse analysis retains the network node configuration, converting the watercourse edges into pressure relationship edges, redefining edge weights, and constructing a pressure feedback network. An improved cascading failure model is then employed to analyse the patterns of reverse risk propagation. The research framework is shown in Fig 1. The specific configuration of the network and model is detailed in this section.

**Fig 1:**
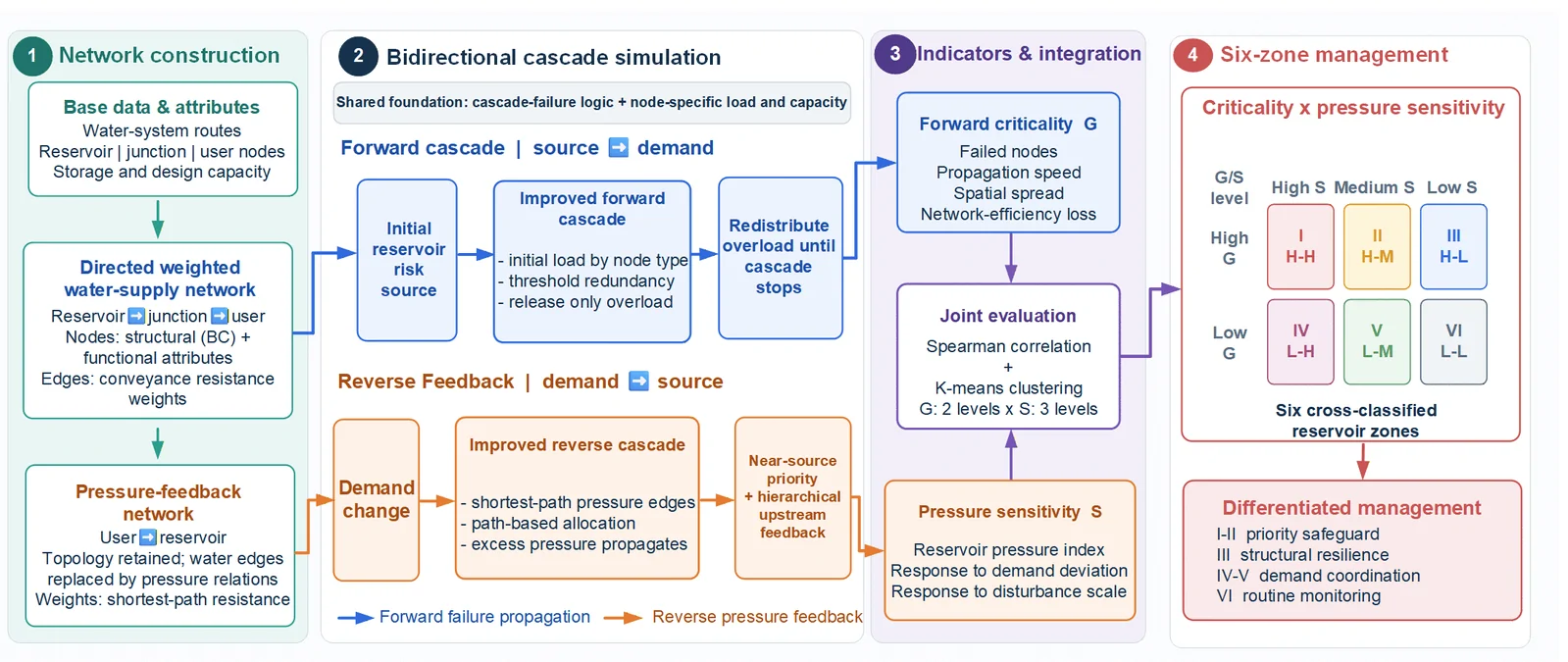
Research framework.

### Network construction

#### Water supply network.

The network comprises three types of nodes: reservoir nodes, watercourse junction nodes and user nodes. Reservoir nodes were selected from official data published by Henan Province on large and medium sized reservoirs to represent the region’s control nodes, totaling 149 reservoirs, comprising 28 large reservoirs and 121 medium sized reservoirs. Natural watercourse junction nodes are logical nodes used to represent the physical connections between rivers, canals and water transmission and distribution lines, totaling 981. User nodes are selected from each city’s most representative water treatment plants that draw their water from surface sources, serving to represent the functional endpoints of the water network system, totaling 153. Edges are used to describe the connections between nodes. Hydrographic vector data were obtained from OpenStreetMap (OSM) through the BBBike extract service. The hydrographic vector data were then imported into BIGEMAP Pro for preprocessing and editing, and missing watercourse connections were subsequently supplemented manually. The completed watercourse data was then exported, resulting in a total of 1,434 watercourse groups. Based on the acquired watercourse data and the basic data for the three types of nodes, a topological network model of Henan Province’s water network was constructed using PyCharm 25.3.1, as shown in Fig 2. The data used to construct the network are provided in S1 File, and the corresponding Python code is provided in S2 File.

**Fig 2:**
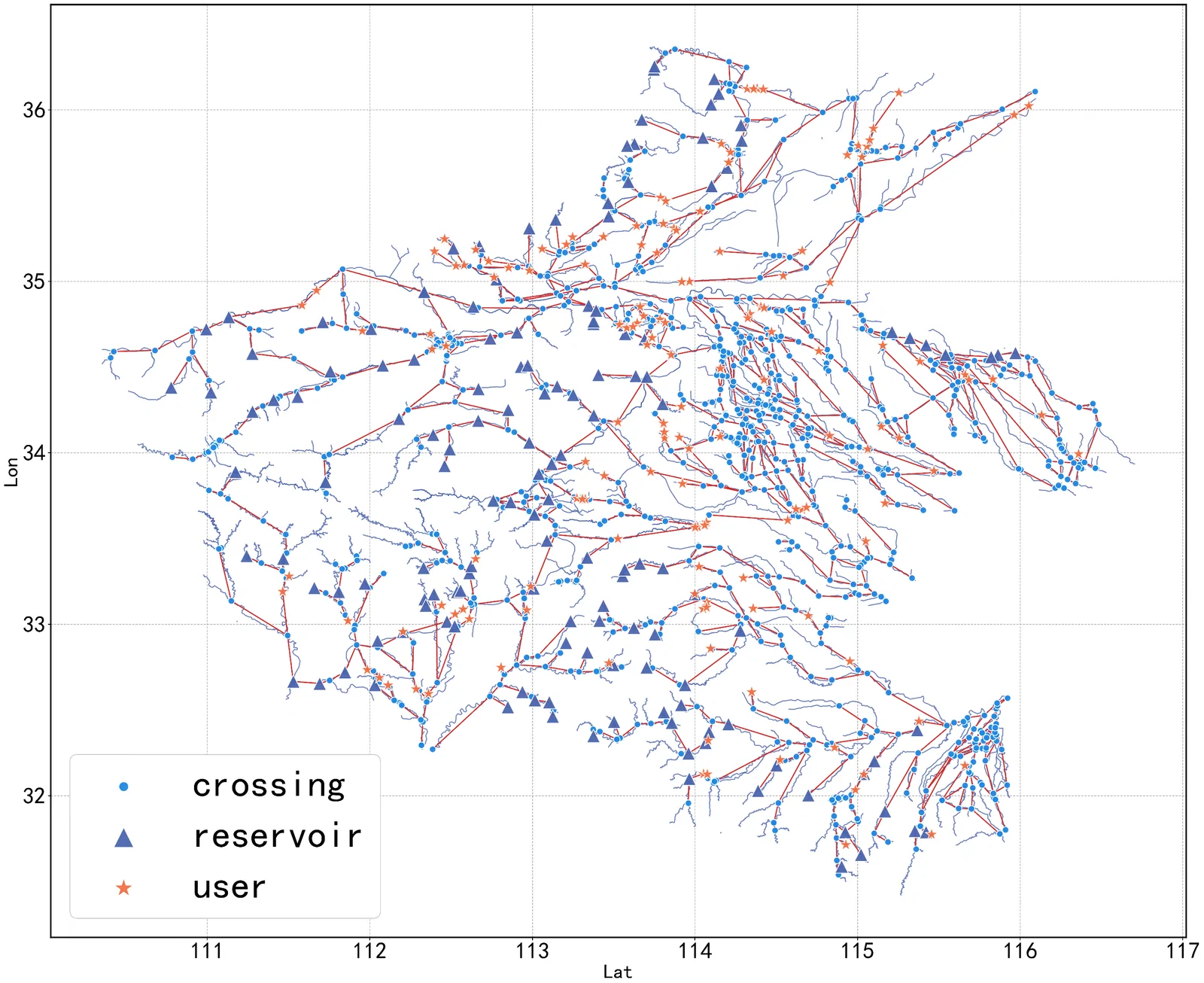
The Henan Province water network. Hydrographic vector data were obtained from OpenStreetMap through the BBBike extract service. Contains information from OpenStreetMap, which is made available under the Open Database License.

1) Node attribute configuration: Attribute information corresponding to the role and function of each node type was assigned. The functional attribute was set to reservoir storage capacity for reservoir nodes, was set to the design capacity of the water treatment plant for user end nodes, and for junction nodes, no functional attribute was assigned, as these nodes represent network connectivity logic. Structural attribute was configured for all three node types in accordance with network metrics.
2) Edge attribute configuration: Given that the ability of risk to propagate varies across different water conveyance channels, the edge weight is defined as the resistance to risk propagation,which means the greater the edge weight, the greater the resistance to risk propagation in that channel, and the more difficult it is for risk to propagate.

$$\begin{array}{c} \omega_{ij}^{\text{1}}=l_{ij}^{\text{1}}\cdot \theta_{\text{1}} \end{array}$$ (1)

$$\begin{array}{c} \omega_{ij}^{\text{2}}=l_{ij}^{\text{2}}\cdot \theta_{\text{2}} \end{array}$$ (2)

Here, $\omega_{ij}^{\text{1}}$ and $\omega_{ij}^{\text{2}}$ denote edge weights($\text{m}\cdot \text{s}\cdot {\text{m}}^{-\text{1}/\text{3}}$), while $l_{ij}^{\text{1}}$ and $l_{ij}^{\text{2}}$ denote edge lengths. The roughness coefficient for natural watercourses is $\theta_{\text{1}}$, and that for water diversion and supply projects is $\theta_{\text{2}}$. In functional landscape modellingt [34], least-cost distance is jointly determined by spatial distance and relative movement cost.Based on this, the edge weights are defined as the resistance to risk propagation, and this value is determined by the spatial distance and the propagation resistance of the corresponding water delivery paths.

Moreover, the edge weight resistance coefficient θ here refers to the Manning’s resistance coefficient. The Manning’s resistance coefficient is an empirical parameter in the Manning formula, used to quantify the frictional resistance on the surface of channels or rivers, units of ${\text{s}\cdot \text{m}}^{-\text{1}/\text{3}}$. A larger θ value indicates greater flow resistance. Because the study covers a province-wide water network, consistent reach-specific data on channel geometry, bed material, vegetation, maintenance condition, discharge, and water level were unavailable for all network edges. Representative roughness values were therefore assigned according to channel type. Manning’s roughness coefficient was set to $\theta_{\text{1}}=\text{0}.\text{0}\text{3}\text{2}{\text{ s}\cdot \text{m}}^{-\text{1}/\text{3}}$ for natural watercourses, which lies within the commonly reported range of 0.025−0.033 for natural streams $\theta_{\text{2}}=\text{0}.\text{0}\text{1}\text{7}\;{\text{s}\cdot \text{m}}^{-\text{1}/\text{3}}$ for artificial pipeline, which generally ranges from 0.010−0.020,considering the presence of joints, bends, wall aging, and minor sediment deposition [35].

Based on the above definitions of nodes, edges and their corresponding attributes, a directed weighted topological network $\text{G}=\left\{\text{N},\text{E},\text{A},{\text{W}}_{\text{N}},{\text{W}}_{\text{E}}\right\}$ is constructed, where $\text{N}=\left\{{\text{N}}_{\text{1}},{\text{N}}_{\text{2}},{\text{N}}_{\text{3}}\right\}$ represents the set of network nodes (${\text{N}}_{\text{i}}$ denote river confluence nodes, reservoir nodes and user end nodes respectively). $\text{E}=\left\{{\text{e}}_{\text{ij}}=({\text{v}}_{\text{i}},{\text{v}}_{\text{j}})\right\}$ represents the set of edges between nodes in the water network. $\text{A}=\left\{{\text{a}}_{\text{ij}}\right\}$ represents the network’s adjacency matrix, where there is an edge ${\text{a}}_{\text{ij}}=\text{1}$ between node  i and node j, and no edge ${\text{a}}_{\text{ij}}=\text{0}$. ${\text{W}}_{\text{N}}=\left\{{\text{W}}_{\text{N1}},{\text{W}}_{\text{N2}},{\text{W}}_{\text{N3}}\right\}$ represents the set of functional attribute values for nodes. ${\text{W}}_{\text{N}}$ denotes the functional attribute values for river confluence nodes (as river confluence nodes are used to characterize the network’s connectivity properties, no functional attributes are assigned to them, i.e., ${\text{W}}_{\text{N1}}=\left\{\emptyset \right\}$). ${\text{W}}_{\text{N2}}$ denotes the set of reservoir capacities. ${\text{W}}_{\text{N3}}$ denotes the set of design capacities for user end nodes. ${\text{W}}_{\text{E}}=\left\{\omega_{\text{ij}}\right\}$ represents the set of edge weights. The term ‘directed’ here is defined according to the direction of risk propagation: forward propagation refers to risk spreading from the reservoir nodes at the water source, through the junction nodes, and then to the user nodes at the water consumption end,on the contrary, reverse propagation refers to risk spreading from the user nodes back to the reservoir nodes.

#### Pressure feedback network.

A pressure feedback network is based on the water network topology, but replaces the original water system edges with pressure relationship edges. The possible node connection scenarios are shown in Table 1. The basic node settings remain unchanged, but the role of junction nodes has changed, junction nodes are not under pressure, but serve to distinguish between different connection paths.

**Table 1:** Node connection scenarios.

Basic Scenarios for Node Connections	Explanation
{U-−(C)-−R}	A user node is connected to a reservoir node (n≥0) vian junction nodes
{U-−(C)-−R1-−(C)-−R2}	A user node has multiple parent reservoir nodes
{U1-−U2-−(C)-−R}	Multiple user nodes are on the same pressure feedback edge U1 When bothU2 experience demand fluctuations, the pressure loads are cumulative If only one generates a demand fluctuation, the other is handled according to the junction node

Property settings: The properties of the nodes remain unchanged. The only difference is that the edges representing pressure relationships are assigned the shortest path edge weights. The shortest path edge weights are set to $\omega_{\text{ab}}=\text{min}\sum_{\text{i}\in {\text{E}}_{\text{ab}}}\omega_{\text{ai}}$, where $\omega_{\text{ai}}=\sum_{\text{j}\in {\text{E}}_{\text{ab}}}{\text{L}}_{\text{ij}}$. The set of paths from the user node a to the reservoir node b is ${\text{E}}_{\text{ab}}$. $\omega_{\text{ai}}=\sum_{\text{j}\in {\text{E}}_{\text{ab}}}{\text{L}}_{\text{ij}}$ denotes the total length of the ith path in the path set ${\text{E}}_{\text{ab}}$. The length of the path with the smallest total length is set as the edge weight for the edge connecting the user node a to the reservoir node b.

#### Network metrics.

Network metrics are used for the subsequent development of models and the analysis of simulation results.

(1) Shortest path. This refers to the path with the lowest total transmission resistance among the multiple reachable paths between a pair of nodes. As edge resistances vary within the water network and risk propagates preferentially along paths of lower resistance, defining the shortest path in terms of minimum total resistance better reflects the dominant transmission pathways for risk in the actual water network. The metric is defined as follows:

$$\begin{array}{c} {\text{d}}_{\text{st}}{=}_{{\text{p}}_{\text{st}}}^{\text{min}}\sum_{(\text{i},\text{j})\in {\text{p}}_{\text{st}}}\omega_{\text{ij}} \end{array}$$ (3)

$p_{st}$ This refers to all paths from s to t, where $\omega_{ij}$ represents the resistance from node i to node j.

(2) Intermediary centrality. This measures the extent to which a ‘given node’ lies on the shortest paths between other ‘node pairs’. Specifically, it is the proportion of the total number of shortest paths $\sigma_{st}$ from s to t that pass through node i. If a node frequently appears on the shortest paths between different node pairs, this indicates that the node plays a stronger role in path relaying and transmission within the network, and thus has a higher intermediary centrality. In the context of water network systems, nodes with high intermediateness centrality are typically located along backbone channels or at key junctions, and are more likely to play an amplifying and propagating role in the transmission of risks.

$$\begin{array}{c} B_{i}=\sum_{s\text{≠}i\text{≠}t}\frac{\sigma_{st}(i)}{\sigma_{st}} \end{array}$$ (4)

(3) Network efficiency. This measures the efficiency with which effective connections are maintained between any two nodes in the network. A higher value for this indicator indicates that the water network as a whole is more efficient at maintaining effective connections.

$$\begin{array}{c} E=\frac{\text{1}}{N(N-\text{1})}\sum_{\text{1}\text{≠}J}\frac{\text{1}}{d_{ij}} \end{array}$$ (5)

Here, $\frac{\text{1}}{d_{ij}}$ represents the connectivity efficiency between a pair of nodes. The lower the path resistance $d_{ij}$ between a pair of nodes, the higher the connectivity efficiency between them. Conversely, as path resistance increases, connectivity efficiency decreases. When a pair of nodes is unreachable, $\frac{\text{1}}{d_{ij}}=\text{0}$. The network efficiency indicator is obtained by taking the average of the connectivity efficiencies for all pairs of nodes in the network.

### Improved cascading failure model

#### Improved forward cascading failure model.

Unlike the traditional cascade failure model: when a reservoir node serves as the initial risk source node, the nodes in the water network carry a load during normal operation. Therefore, all three types of nodes are assigned an initial risk load at the start. Secondly, considering that nodes retain a certain degree of risk resistance even after failure, only the portion exceeding the carrying capacity is released when the risk load of a failed node is discharged.The forward cascading failure model is configured as follows:

(1) Initial risk load of nodes${\text{L}}_{\text{i}}$

To reflect the structural and functional differences between node types in risk propagation, different initial risk loads are set for each of the three node types. Junction nodes possess only structural attributes, and their initial risk load is determined by the normalized value of their intermediary centrality. Reservoir nodes and user nodes possess both structural and functional attributes, and their initial load is calculated as the weighted sum of the indicators for these two attributes.

1) Interconnection nodes

$$\begin{array}{c} L_{\text{1}i}={\hat{B}}_{\text{1}i} \end{array}$$ (6)

where $\hat{B_{i}}=\frac{{\text{2}B}_{i}}{\left(N-\text{1})(N-\text{2}\right)}$, representing the normalized values of the intermedia centrality of the three types of nodes.


$$\begin{array}{c} L_{\text{2}i}=\lambda {\hat{V}}_{i}^{*}+(\text{1}-\lambda ){\hat{B}}_{\text{2}i} \end{array}$$ (7)

2) Reservoir nodes

As there is a significant disparity in reservoir capacity (V) between ultra large and medium sized reservoirs in this province, and to prevent the functional attributes of reservoirs with smaller capacities from being underrepresented by simple normalization, this paper applies logarithmic compression to the reservoir capacity data:


$$\begin{array}{c} {\hat{V}}_{i}^{*}=\frac{log(\text{1}+V_{i})-min(log(\text{1}+V))}{max(log(\text{1}+V))-min(\text{log}(\text{1}+V))} \end{array}$$ (8)

$$\begin{array}{c} L_{\text{3}i}=\mu {\hat{D}}_{i}^{*}+(\text{1}-\mu ){\hat{B}}_{\text{3}i} \end{array}$$ (9)

3) User nodes

Similarly, the design capacityD is processed as follows:


$$\begin{array}{c} {\hat{D}}_{i}^{*}=\frac{log\left(1+D_{i}\right)-min(log(1+D))}{max\left(log(1+D)\right)-min(log(1+D))} \end{array}$$ (10)

(2) Node Risk Thresholds ${\text{C}}_{\text{i}}$

Node risk thresholds are used to characterize the maximum risk value that a node can withstand under the combined effects of risk shocks and sustained transmission pressures. The risk threshold is set as a redundancy capacity equal to a percentage $\alpha_{i}$ added to the initial load. Different redundancy coefficients are set to reflect differences in carrying capacity ($\alpha_{\text{2}}$>$\alpha_{\text{3}}$>$\alpha_{\text{1}}$): junction nodes $C_{\text{1}i}=(\text{1}+\alpha_{\text{1}})L_{\text{1}i}$; reservoir nodes $C_{\text{2}i}=(\text{1}+\alpha_{\text{2}})L_{\text{2}i}$; user nodes $C_{\text{3}i}=(\text{1}+\alpha_{\text{3}})L_{\text{3}i}$.

(3) Determination of the initial risk source and release of the load at failed nodes

For forward propagation, the reservoir node is selected as the initial risk source node. Let the initial risk source node be s, with an initial risk load of $L_{s}^{\text{0}}$ and a risk threshold of $C_{s}$. Taking external disturbances into account, the impact load on the initial risk source is defined as:


$$\begin{array}{c} {\tilde{L}}_{s}(\text{0})=\rho L_{s}^{\text{0}} \end{array}$$ (11)

Where $\rho_{\text{1}}$ is the external impact coefficient $\rho_{\text{1}}>\text{1}+\alpha_{\text{1}}$, used to ensure that the initial risk source enters an overloaded state following an external disturbance.

Unlike previous cascade failure propagation models, which assume that a node releases its entire load upon failure, nodes in a water network retain a certain level of risk resistance after failure. Consequently, the load released by a failed node is defined as the overload:


$$\begin{array}{c} \Delta L_{s}(\text{0})={\tilde{L}}_{s}(\text{0})-C_{s} \end{array}$$ (12)

Node s, as the failed node in layer 0, transmits its overload portion to adjacent nodes in accordance with the risk redistribution principle.

(4) Risk redistribution rules

The transmission probability ${\text{p}}_{\text{ij}}=\frac{\frac{\text{1}}{\omega_{\text{ij}}}}{\sum_{\text{k}\in \text{out}(\text{i})}\frac{\text{1}}{\omega_{\text{ik}}}}$ links the probability of risk transmission to the connection resistance between nodes. The lower the resistance, the easier it is for risk to propagate along that path. Based on this, the overload risk released by the failed node is distributed to neighbouring nodes according to the transmission probability. The allocated amount $\Delta L_{ij}=p_{ij}\cdot (L_{i}(t)-C_{i})$ is used to update the current risk load $L_{j}(t+\text{1})=L_{j}(t)+\sum_{i\in in(j)}\Delta L_{ij}(t)\;$ of neighbouring nodes after they have accepted the additional risk load. If their updated risk load exceeds their own threshold, i.e., $L_{j}(t+\text{1})>C_{j}$, the node $L_{j}(t+\text{1})$ fails.Proceed to the next propagation layer to continue risk release and propagation.

#### Performance indicators for forward cascade failure.

(1) Number of Final Failed Nodes

$$\begin{array}{c} N_{f}=|F| \end{array}$$ (13)

The number of final failed nodes is used to characterize the scale of node failures caused by risk propagation. The higher the indicator value, the more severe the consequences of the cascading failure. It is defined as follows:

N denotes the total number of initial network nodes. The set of failed nodes upon completion of the cascade failure process is F, and $N_{f}$ denotes the number of final failed nodes.

(2) Number of propagation layers and propagation speed

Let the initial risk source node be denoted as layer 0. If, after t rounds of propagation, the set of newly failed nodes is $F_{t}$, and no new failed nodes appear after t+1 rounds, the cascading failure process terminates. Supposing that a total of T rounds of propagation have occurred at the time of termination, the number of propagation layers is:


$$\begin{array}{c} H=T \end{array}$$ (14)

This metric reflects the propagation depth of cascading failures within the network. The larger the value of H, the further the risk can propagate along the network structure, and the more pronounced the cascading failure effect of the network.


$$\begin{array}{c} V=\frac{\text{1}}{T}\sum_{t=\text{1}}^{T}n_{t}=\frac{N_{f}-N_{\text{0}}}{T} \end{array}$$ (15)

Here,$n_{t}$ represents the number of new failure nodes in the tth round of propagation, while $N_{\text{0}}$ denotes the number of initial failure nodes. When only one initial risk source is selected, $N_{\text{0}}=\text{1}$. V denotes the average number of new failure nodes added at each propagation step during the cascading failure process. The higher this metric, the more rapidly risk propagates through the network, and the more likely the system is to experience widespread chain failures following a disturbance.

(3) Spatial Propagation Range

The spatial propagation range metric is introduced to characterize the spatial extent of the impact of cascading failures. Let the set of nodes affected during the cascading failure process be denoted as S, which includes the set of ultimately failed nodes F as well as nodes whose risk load has increased during propagation. Based on the spatial coordinates of the affected nodes, the area of the outer envelope $A_{s}$ is used to characterize the spatial coverage of risk propagation. Given that water network nodes are distributed in a strip along the river channel, a concave envelope is used to fit the actual diffusion range of the affected nodes:


$$\begin{array}{c} A_{s}=Area(AlphaShape(S,\alpha )) \end{array}$$ (16)

AlphaShape(S,α) This represents the concave envelope constructed from the set of affected nodes S under the shape control parameter α.

(4) Loss of Network Efficiency

The network efficiency loss metric is used to characterize the impact of cascading failures on network connectivity efficiency. The higher the network efficiency loss metric, the more pronounced the decline in overall network connectivity following node failure, and the more severe the degradation of system functionality. The metric is defined as follows:


$$\begin{array}{c} L_{E}=\frac{E_{\text{0}}-E_{f}}{E_{\text{0}}} \end{array}$$ (17)

Where $E_{\text{0}}$ represents the initial network efficiency, and $E_{f}$ represents the residual network efficiency following the cascading failure.

(5) Comprehensive Indicator for Evaluating Node Criticality

To analyse critical initial risk source nodes in the network, a composite indicator G is defined to represent the criticality of different nodes within the reservoir network. As each indicator reflects the results of risk propagation from a different perspective, and to prevent any single result from dominating the evaluation of node criticality, it is assumed that all indicators carry equal weight [36], where $\hat{N_{f}},\hat{V},\hat{A_{s}},\hat{L_{E}}$ represents the normalized values of the four indicators respectively.


$$\begin{array}{c} G=(\hat{N_{f}}+\hat{V}+\hat{A_{s}}+\hat{L_{E}})/\text{4} \end{array}$$ (18)

#### Improved reverse cascading failure model.

The improvements to the traditional cascade failure model in this model are primarily reflected in the load redistribution rules. The traditional model relies on direct topological connections between nodes to distribute the load released by a failed node to neighbouring nodes according to transmission probabilities. The improved cascade failure model first identifies several reachable connection paths between the risk node and potential load bearing nodes, then retains the path with the lowest connection resistance as the effective transmission path for the stress load, and finally distributes the load according to the transmission probabilities between nodes.

In reality, as the water demand at the user end increases, the user end will first look for water supply from nearby reservoir nodes. If the nearby reservoir nodes cannot meet their demands, they will then transfer the water supply to distant reservoir nodes. This is the actual situation of water resource management in the water network system. In addition, the risk back propagation considered in this study is a simplified management abstraction and does not specifically account for the effects of changes in pressure, flow, hydraulic resistance, or equipment operating modes on this process. Based on this, this paper designs an improved cascading failure model to simulate this process. The deviation of the user node’s demand leads to the generation of the initial risk source, which then triggers the cascading failure; after the failure, the pressure load of the nodes is first transferred to nearby reservoir nodes. If the received load exceeds the threshold of the reservoir node, the excess load will be allocated to other distant reservoir nodes.The specific model settings are as follows:

(1) Initial pressure load. When the user node acts as the initial risk source, it receives signals of demand fluctuations and converts these into a pressure risk load:

$$\begin{array}{c} L_{Ui}^{B}(\text{0})={\gamma \hat{D}}_{i}^{*}(\text{1}+|\beta_{i}|) \end{array}$$ (19)

Where γ represents the conversion coefficient for transforming demand deviation into pressure, ${|\beta }_{i}|$ represents the magnitude of demand deviation, and ${\hat{D}}_{i}^{*}$ represents the logarithmically compressed value of the user node’s design capacity.

(2) Pressure threshold for reservoir nodes. Junction nodes are not subject to pressure and exist solely as connection logic. Only reservoir nodes are subject to pressure, and the pressure threshold is set as follows:

$$\begin{array}{c} T_{R}=\sigma {\hat{V}}_{i}^{*} \end{array}$$ (20)

The pressure threshold for a reservoir node is related to its storage capacity, ${\hat{V}}_{i}^{*}$ represents the logarithmically compressed value of the reservoir node’s storage capacity.

(3) Load redistribution rules

Transmission probability $p_{i}=\frac{\frac{\text{1}}{\omega_{i}}}{\sum_{i\in m}\frac{\text{1}}{\omega_{i}}}$: this can be understood as the allocation ratio of the ith path among the m transmission paths from a user node to a connected reservoir node. The smaller the resistance$\omega_{i}$, the greater the transmission probability, and the easier it is for pressure to propagate along that path.The pressure load received by a reservoir node is$P_{R}=p_{i}\Delta L_{Ui}^{B}(\text{0})$, $\Delta L_{Ui}^{B}(\text{0})=\gamma {\hat{D}}_{i}^{*}{|\beta }_{i}|$. If$P_{R}{<T}_{R}$, the reservoir node can continue to withstand the pressure. If $P_{R}{>T}_{R}$, the node’s pressure exceeds the limit and the node fails. When a reservoir node in close proximity to a user node fails, the pressure is propagated to the upstream reservoir node.

#### Reverse cascading failure result metrics.

(1) Reservoir node pressure bearing index. $I_{R}=\frac{P_{R}}{T_{R}}$, $I_{R}\in$[0,1], $P_{R}$ denote the pressure loads received by the reservoir node, and $T_{R}$ denotes the pressure bearing threshold of the reservoir node.
(2) Comprehensive sensitivity index for reservoir node pressure. $S=\sqrt{{\text{S}}_{q}^{\text{2}}+{\text{S}}_{\text{k}}^{\text{2}}}$, where ${\text{S}}_{q}=\text{mean}\frac{{\Delta I}_{R}}{\left|\Delta q_{i}\right|}$ represents the sensitivity index value of the reservoir node pressure index to the demand deviation $\left|\Delta q_{i}\right|$, and ${\text{S}}_{\text{k}}=\text{mean}\frac{{\Delta I}_{R}}{\Delta \text{k}}$ represents the sensitivity index value of the reservoir node pressure index to the disturbance scale Δk. The composite sensitivity index S is used to measure how rapidly the reservoir’s stress index changes overall in response to variations in both the ‘magnitude of demand deviation’ and the scale of disturbed users. A higher value indicates that the reservoir node is more sensitive and more susceptible to stress.

## Analysis of model simulation results

Based on the aforementioned topological network model of Henan Province’s water network, a simulation of bidirectional risk propagation and cascading failure was conducted.

The parameter values were determined according to the attribute differences among node types, practical engineering operating characteristics, and related cascading failure models [3,34,37]. The risk-threshold redundancy coefficients $\;\alpha_{\text{1}},\alpha_{\text{2}},\;\alpha_{\text{3}\;}\;$ correspond to junction nodes, reservoir nodes, and user nodes, respectively. Under actual engineering operation, beneficial storage generally accounts for approximately 30%–50% of total reservoir storage, whereas the reservoir storage attribute used in this study is the total storage capacity. Therefore, $\;\alpha_{\text{1}}=\text{0}.\text{1},\alpha_{\text{2}}=\text{0}.\text{4},\alpha_{\text{3}}=\text{0}.\text{3}$ were adopted. The external impact coefficient was set to ρ=1.5. When $\alpha_{\text{2}}=\text{0}.\text{4}$, the risk threshold of the initial reservoir risk source is $\text{1}.\text{4}{\text{L}}_{\text{s}}\left(\text{0}\right)$, while its disturbed risk load is $\text{1}.\text{5}{\text{L}}_{\text{s}}\left(\text{0}\right)$. This setting ensures the failure of the initial risk source and initiates cascading propagation while avoiding an excessively large impact load. The parameters λ=0.8 and μ=0.7  represent the weights of the functional attributes in the initial risk loads of reservoir nodes and user nodes, respectively, indicating that reservoir storage capacity and design capacity have greater effects on the initial risk loads than structural attributes. In the forward model,${\;|\beta }_{\text{i}}|\;$represents the demand deviation and was varied within (0,0.25]. The conversion coefficient from demand fluctuations to pressure risk loads was set to γ=1, indicating that the differential water volume caused by demand fluctuations is fully converted into a pressure risk load. The conversion coefficient from normalized reservoir storage capacity to the pressure threshold was set to σ=1, such that the normalized reservoir storage capacity directly represents the relative pressure-bearing capacity of reservoir nodes.

### Forward cascading failure simulation

#### Simulation results.

The simulation results for cascading failures with reservoir nodes as the initial risk source are detailed in Figs 3–5. As shown in Fig 3, the risk propagation triggered by reservoir nodes exhibits a head-concentration phenomenon, with a small number of nodes experiencing large scale failures while the majority suffer smaller scale failures. The majority of both the failed nodes and the affected but non-failed nodes are watercourse junction nodes. There are variations in the composition of affected nodes following the failure of a reservoir node. Most of the risk is first absorbed and buffered at the confluence nodes before being transmitted to end users. However, end users possess a certain degree of redundancy, manifesting as disturbance rather than immediate failure. Furthermore, a few reservoir nodes exhibit disturbances on other reservoir nodes, indicating that risk propagation also occurs between reservoir nodes.

**Fig 3:**
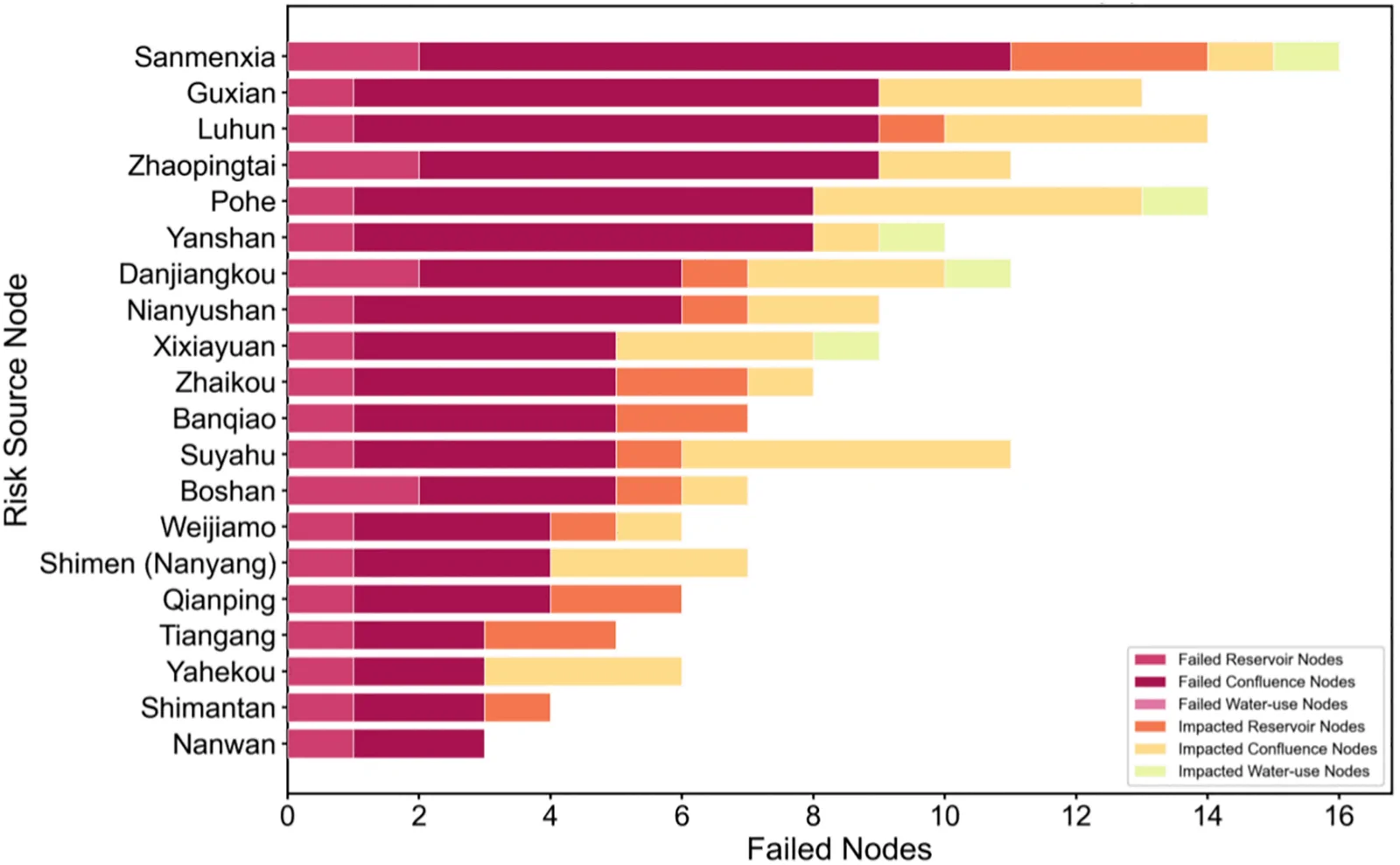
Number of failed nodes.

**Fig 4:**
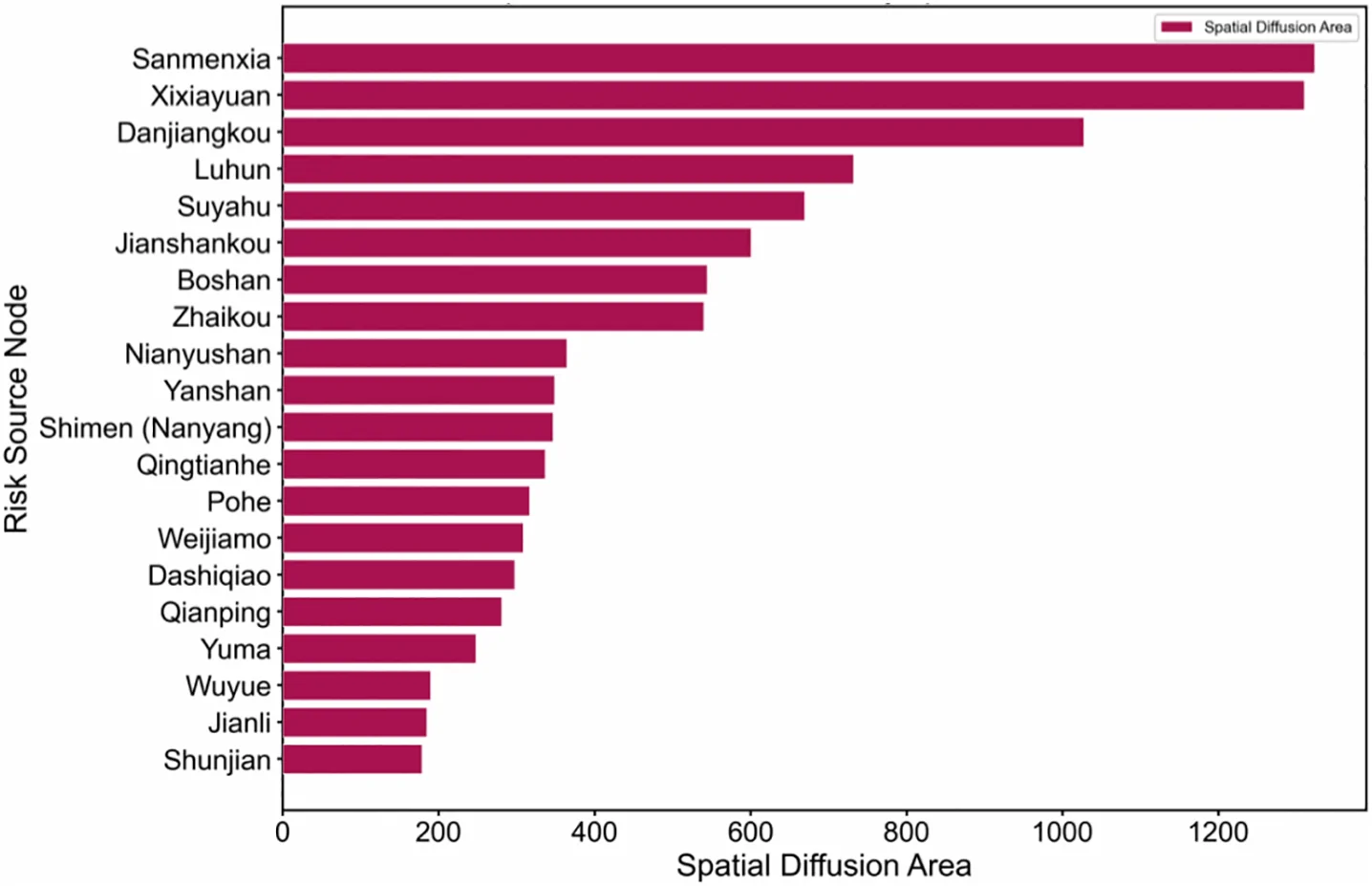
Spatial propagation range.

**Fig 5:**
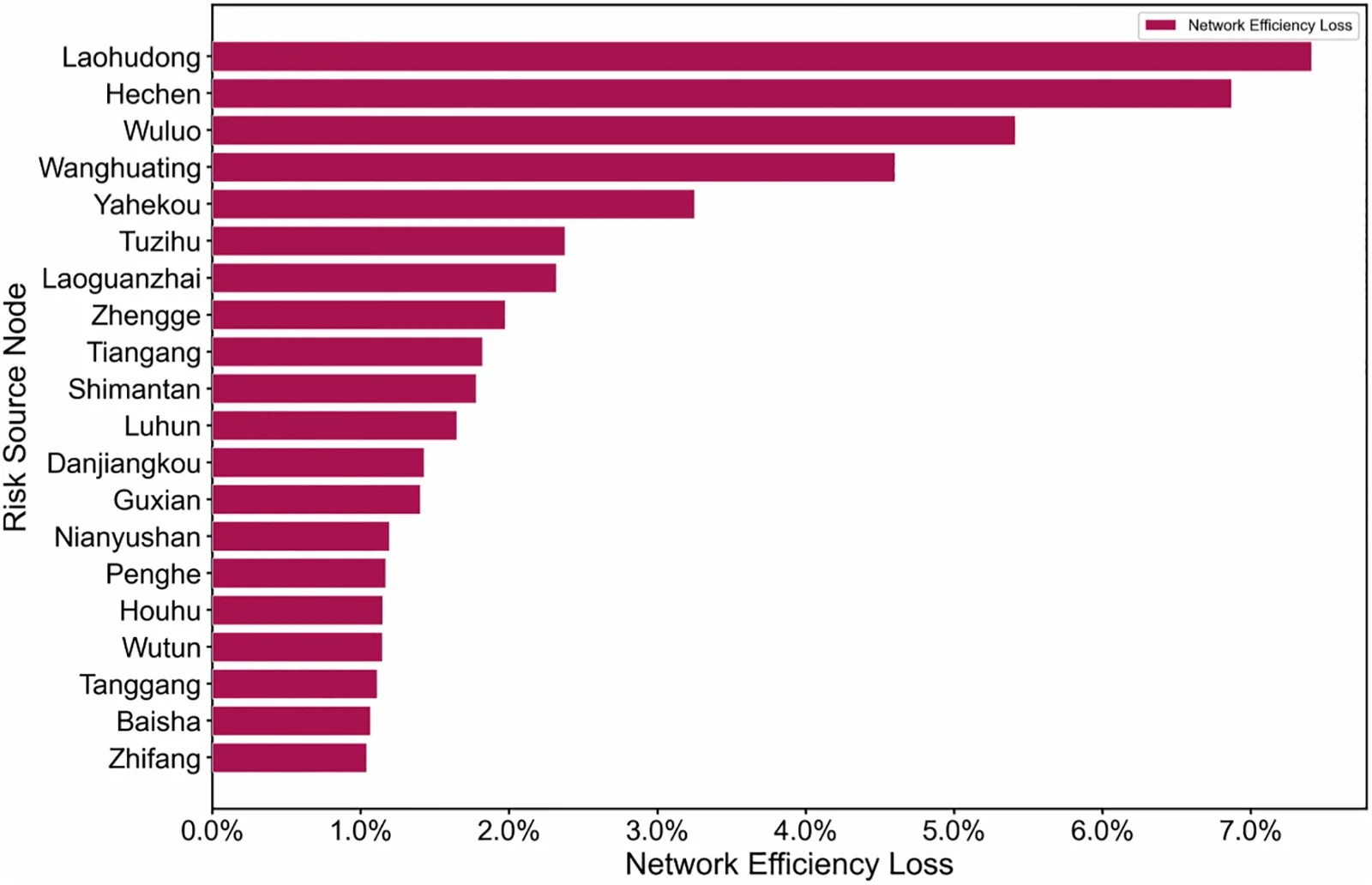
Network efficiency loss.

Cascading failures of reservoir nodes exhibit the characteristic of long distance propagation along the backbone network. As shown in Fig 4, which ranks the top 20 reservoir nodes by spatial propagation range, when nodes such as Sanmenxia Reservoir, Xixia Yuan Reservoir, Danjiangkou Reservoir and Luhun Reservoir act as initial risk sources, the spatial coverage of the failure is relatively extensive. As large reservoirs in Henan Province, these nodes occupy key positions in regional water supply or inter-basin water transfer systems and are connected to multiple downstream nodes. Once a node fails, the risk load propagates over long distances along the main water conveyance channels and trunk and branch lines, thereby exhibiting a large spatial coverage.

Judging from the ranking results in Figs 3 and 4, risk source nodes with a high number of failed nodes are not necessarily the same as those with a large spatial diffusion range. The former are concentrated at nodes with a higher density of adjacent nodes, and their indicators focus on the structural characteristics of the network. The latter are concentrated at nodes in the backbone network, at upstream control points, and at nodes responsible for cross regional water conveyance and distribution, with their indicators covering both structural and functional characteristics of the water network.

Judging from the results on network efficiency loss (Fig 5), the impact of different reservoir risk sources on the overall connectivity of the water network varies, and also exhibits a head heavy distribution, with a maximum efficiency loss of approximately 7.41%. The relatively limited loss of network efficiency does not contradict the large number of failed nodes or the extensive spatial propagation observed in some scenarios, because these indicators reflect different dimensions of cascading failure. A large spatial propagation range indicates that risk can travel over a long geographical distance, but does not mean that a large number of global shortest paths are disrupted. Similarly, a large number of failed nodes does not necessarily cause a substantial efficiency loss, because nodes at different topological positions make unequal contributions to global connectivity. Therefore, the results indicate that the cascading failures caused by individual reservoir nodes have a relatively limited impact on the shortest-path structure of the water network.

#### Analysis of propagation patterns.

As the second largest reservoir in Henan Province and the ‘master switch’ for water and sediment regulation on the Yellow River, the Sanmenxia Reservoir plays a crucial role among the reservoir nodes in Henan Province. Furthermore, when the Sanmenxia Reservoir is designated as the initial risk source node, the cascade failure simulation results reveal a higher number of node failures and a larger spatial propagation range. Therefore, the Sanmenxia Reservoir node was selected to analyse the patterns of risk propagation.

The risk propagation simulation process and results are shown in Figs 6 and 7. The final number of failed nodes was 11, with a network efficiency loss of 0.63%, a propagation speed of 1.67 (nodes/step), a spatial propagation range of 1,323.36 km^2^, and a comprehensive criticality index of 0.677.

**Fig 6:**
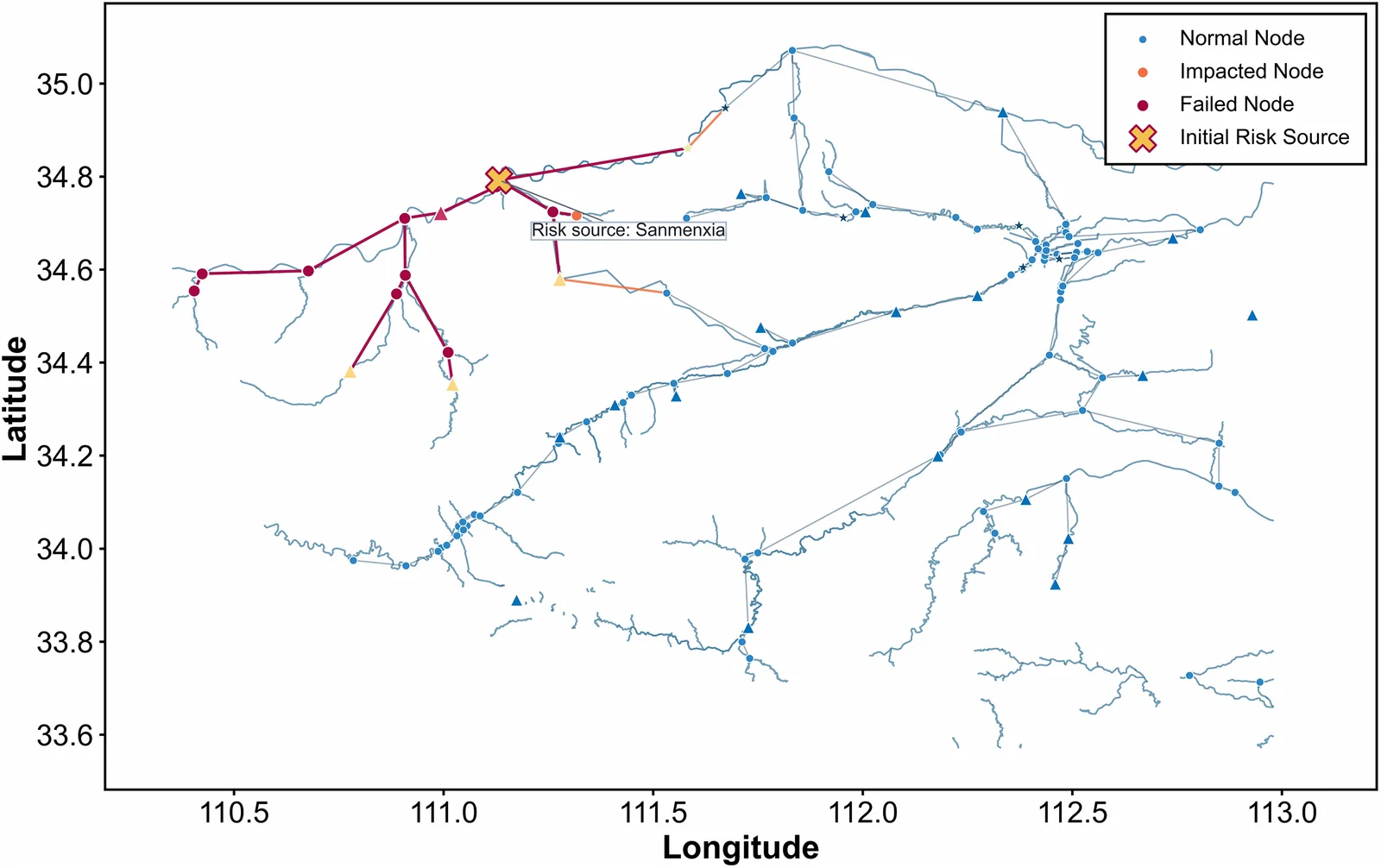
Schematic diagram of the propagation process. Hydrographic vector data were obtained from OpenStreetMap through the BBBike extract service. Contains information from OpenStreetMap, which is made available under the Open Database License.

**Fig 7:**
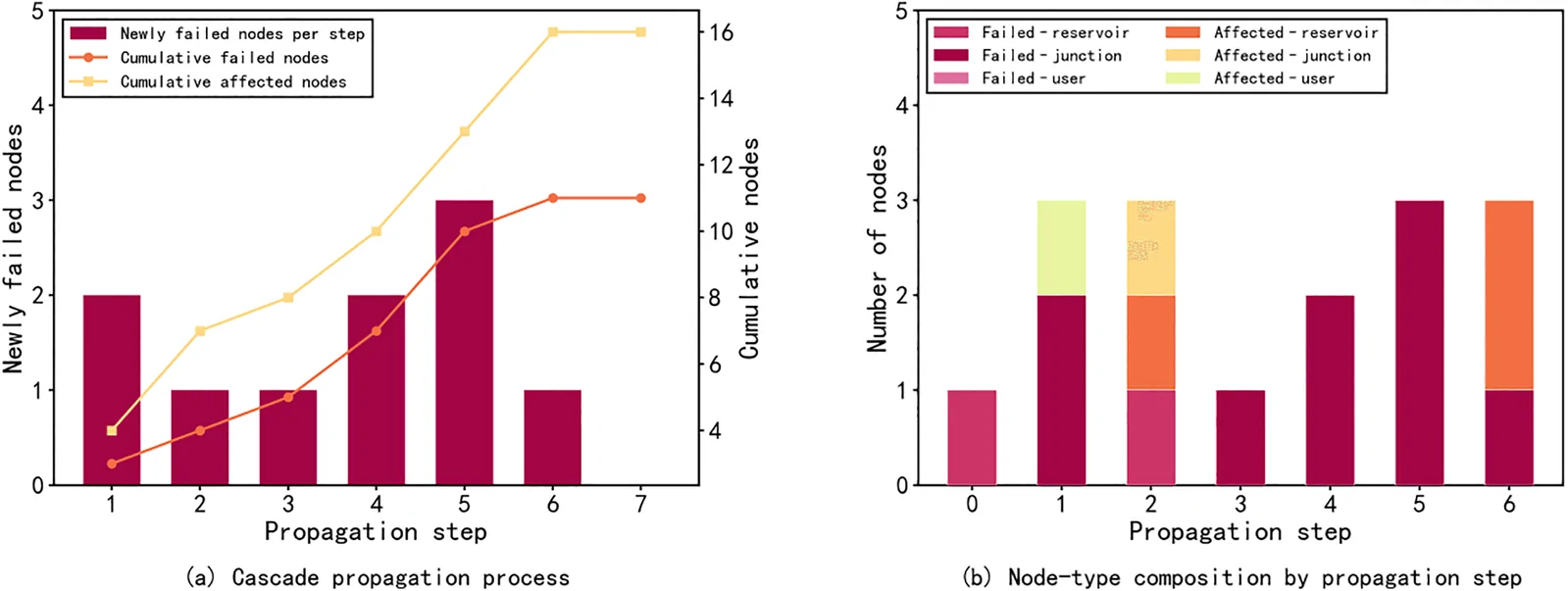
Risk propagation process.

Judging from the risk propagation process and the composition of affected nodes (Fig 7), risk propagation exhibits a temporal evolution characterized by an initial intensification followed by a subsequent weakening. The risk propagation process can be divided into three stages: Initial activation stage – in the first two steps, the number of newly failed nodes is small, and risk propagation is primarily confined to local areas, with a relatively limited scope. Rapid amplification stage – after step 3, the number of newly failed nodes rises significantly, peaking at step 5, indicating that the risk has reached highly connected or critical nodes within the network, and that risk propagation has shifted from initial local transmission to structural amplification. Decay and convergence stage-after step 5, the number of newly failed nodes begins to decline, indicating that the risk propagation capacity is weakening and the process is entering a dissipation phase. The decline in the number of failed nodes at Step 2 in Fig 7-a is corroborated by the composition of the affected nodes (Fig 7-b). During the propagation at Step 2, two reservoir nodes absorbed risk loads exceeding their limits,and one of these nodes exceeded the risk threshold, regarded as a new risk source, continued to release risk loads. By Step 6, the propagation encountered a user node that absorbed the risk load, bringing the diffusion process to an end.

From the cumulative curve of the propagation process, the cumulative number of failed nodes continues to rise but gradually stabilizes in the later stages. In the later stages of risk propagation, the system exhibits a situation where the majority of nodes are affected but do not fail.

When the Sanmenxia Reservoir node acts as the initial risk source, the loss in network efficiency is not significant, but the number of failed nodes and the spatial extent of failure are substantial. The reason is that, following the failure of this reservoir node, a large amount of risk load is released. Even with other reservoir nodes acting as buffers, a significant number of junction nodes and user nodes are still affected. Secondly, as the connections between the reservoir node and other nodes are characterized by long distance backbone links, the propagation pattern when the reservoir node acts as the initial risk source tends to form ‘long chains’, facilitating long distance spatial propagation and consequently resulting in a wider spatial spread.

#### Comparative analysis with the traditional model.

(1) Comparison with Traditional Network Structural Characteristic Analysis

A scatter plot comparing the two rankings is presented in Fig 8. The results show that the ranking of reservoir nodes based on the composite criticality indicator obtained from the improved forward cascading failure model differs from that derived from the traditional analysis of network structural characteristics. The Spearman correlation coefficient and Kendall’s rank correlation coefficient between the two rankings are 0.299 and 0.230, respectively, indicating only weak rank agreement between the two methods. Moreover, the two Top 20 lists share only two critical reservoirs. In the improved forward cascading failure model for setting the initial load of nodes, both the structural indicators-betweenness centrality, which is the analysis of traditional network structure characteristics, and the node functional attributes are taken into account. This is an improvement over the traditional analysis of node criticality which only considers the network structure.

**Fig 8:**
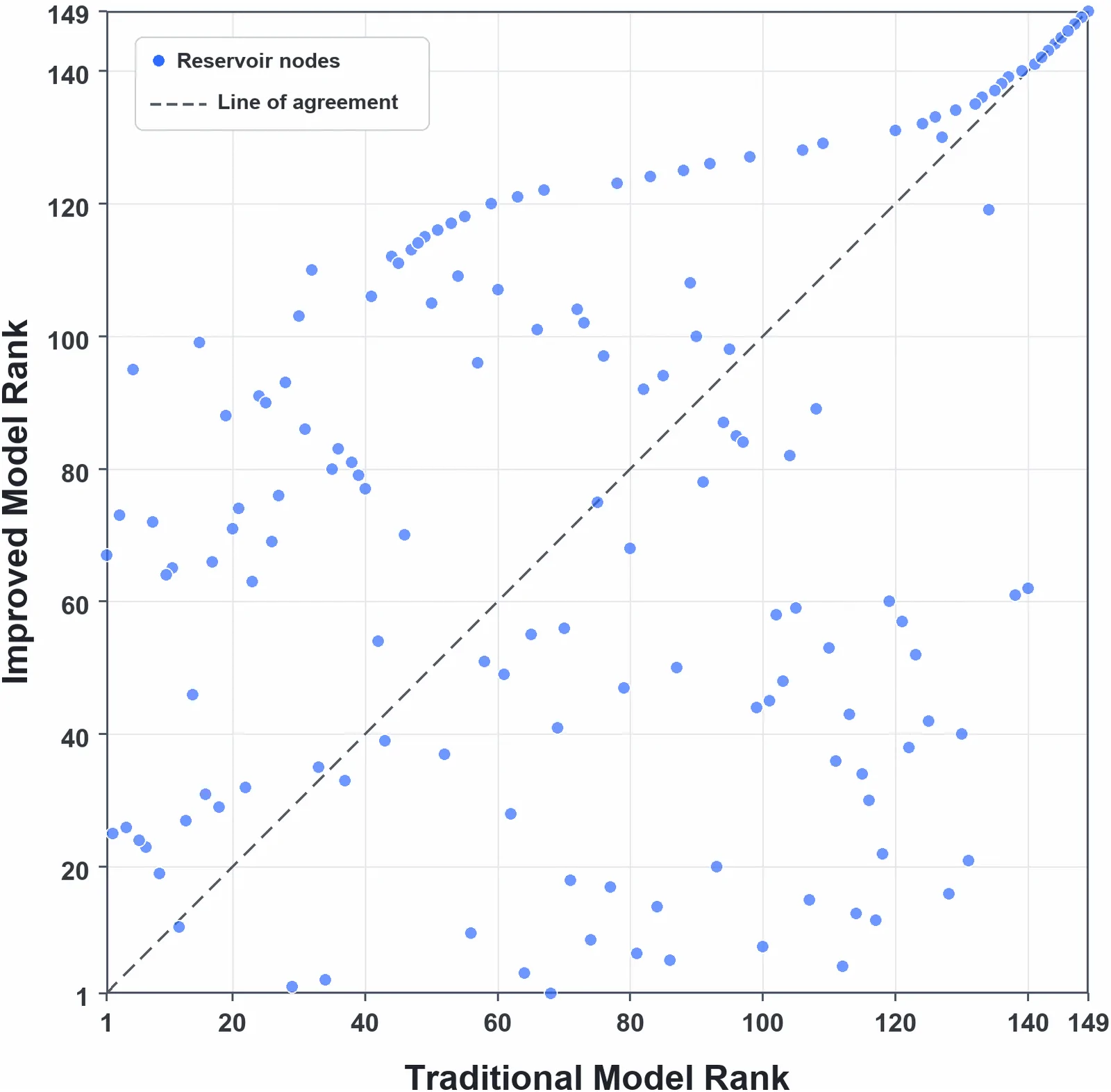
Comparison of reservoir node criticality rank.

(2) Comparison with the Traditional Cascading Failure Model

The traditional model uses only normalized betweenness centrality as the initial node load and therefore assumes that structurally similar nodes have similar initial loads. However, reservoirs are not only topological nodes in a water network but also perform water storage, regulation, and supply functions, and reservoir storage capacity can represent their functional scale. Therefore, the improved model incorporates both betweenness centrality and reservoir storage capacity into the initial-load setting, enabling the initial load to reflect both the structural and functional attributes of nodes and better represent the actual characteristics of reservoir nodes in water-network systems. Second, node failure indicates that the risk load of a node exceeds its load-bearing threshold and does not mean that all of its water storage and regulation capacity disappears. Therefore, compared with the traditional model, which releases the entire load after node failure, the improved model redistributes only the load exceeding the load-bearing threshold, which is more consistent with actual conditions. Except for the initial-load setting and the load-release rule for failed nodes, all other settings of the traditional model remained unchanged.

Based on the criteria of structural similarity and functional heterogeneity, three representative reservoir pairs were selected from the 149 reservoir nodes. Specifically, the difference in betweenness centrality between the two reservoirs in each pair was no greater than 5%, whereas their storage capacities differed by more than a factor of five. The composite criticality rankings obtained using the traditional cascading failure model and the improved forward cascading failure model were then compared, as shown in Table 2.

**Table 2:** Comparison between the traditional and improved cascading failure model.

Reservoir pair	Difference in betweenness centrality (%)	Storage capacity ($10^{8}m^{3}$)	Storage capacity ratio	traditional model		proposed model	
				rank	Rank difference	rank	Rank difference
Nianyushan	0.00	9.16	25.11	56	0	7	101
Tiefosi		0.3648		56		108	
Zhengge	0.00	0.2937	20.70	43	3	24	73
Panshitou		6.08		46		97	
Majiayan	0.00	0.2795	20.89	67	7	54	37
Qianping		5.84		74		17	

As shown in Table 2, the traditional cascading failure model produced identical or similar criticality rankings for reservoirs with similar structural characteristics, even when their functional scales differed substantially. In contrast, by incorporating reservoir storage capacity into the initial-load definition, the improved model effectively distinguished the functional heterogeneity of these reservoirs. Nianyushan and Tiefosi reservoirs had the same betweenness centrality of 0.0072, namely the betweenness centrality differences(Δ) is zero, but differed in storage capacity by a factor of 25.11. Both reservoirs were ranked 56th by the traditional model, whereas they were ranked 7th and 108th separately by the improved model. Similarly, the traditional-model rank differences ($\Delta_{\text{1}}$) for the Zhengge-Panshitou and Majiayan- Qianping pairs were only 3 and 7 positions separately, whereas the corresponding differences ($\Delta_{\text{2}}$) under the improved model increased to 73 and 37 positions separately. These results demonstrate that the improved model has a stronger capability to differentiate reservoir nodes as it more comprehensively reflects the characteristics of the reservoir node in improved cascading failure model.

It should be emphasized that Panshitou and Qianping reservoirs had the same betweenness centrality of 0.0143 and similar storage capacities of $\text{6}.\text{08}\cdot {\text{10}}^{\text{8}}$ and $\text{5}.\text{84}\cdot {\text{10}}^{\text{8}}{\text{m}}^{\text{3}}$, but were ranked 97th and 17th by the improved model. This ranking difference arises because reservoir storage capacity enters the model only through the initial-load setting rather than serving directly as the basis for node criticality ranking. After the initial risk source fails, the extent of risk propagation depends on the reachable propagation paths and their resistance, as well as the residual load-bearing capacities of neighboring nodes. This propagation process further determines the cascading failure results. In this study, nodes are ranked by criticality based on the cascading failure results. When Panshitou Reservoir served as the initial risk source, no secondary node failure occurred, and the affected area and network-efficiency loss were only 18.46 km^2^ and 0.001842 separately. In contrast, the failure of Qianping Reservoir resulted in four failed nodes across two propagation layers, with an affected area of 280.63 km^2^ and a network-efficiency loss of 0.003754. Therefore, even if two reservoirs have similar betweenness centrality and storage capacity, they may receive different criticality rankings when they produce different cascading failure consequences.

#### Model robustness analysis.

To examine whether changes in model parameters affect the model conclusions, the current parameter setting was used as the baseline scenario, and two alternative parameter scenarios—the high-redundancy, high functional-attribute-weight scenario (the high-value scenario) and the low-redundancy, low functional-attribute-weight scenario (the low-value scenario)—were designed for the robustness analysis. In reality, when one parameter fluctuates, the other parameters in the system will also fluctuate simultaneously. If only one parameter is altered while all other parameters remain unchanged, it may result in a parameter combination that does not reflect actual operating conditions.

The parameter settings for the three scenarios are listed in Table 3. The overload released by the initial risk source was kept constant at $\text{0}.\text{1}L_{s}^{\text{0}}$. $\rho -(\text{1}+\alpha_{\text{2}})=\text{0}.\text{1}$. In addition, because junction nodes have limited inherent resistance to risk, $\alpha_{\text{1}max}=\text{0}.\text{1}$.

**Table 3:** Parameter values.

Category	$\alpha_{1}$	$\alpha_{2}$	$\alpha_{3}$	ρ	λ	μ
Baseline scenario	0.1	0.4	0.3	1.5	0.8	0.7
Low-redundancy, low functional-attribute-weight scenario	0.05	0.3	0.2	1.4	0.7	0.6
High-redundancy, high functional-attribute-weight scenario	0.1	0.5	0.4	1.6	0.9	0.8

The results for the three scenarios are compared in Fig 9, demonstrating that parameter changes affect the scale of cascading failures. Relative to the baseline scenario, the average number of failed nodes, propagation speed, spatial propagation range and efficiency loss in the high-value scenario changed by 3.8%, 6.1%, 1.4% and 1.2%, in the low-value scenario changed by 18.1%, 33.3%, 22.4%, and 22.8%. These results indicate that reducing node redundancy and adjusting the relative weights assigned to structural and functional attributes enhance the cascading propagation effect.

**Fig 9:**
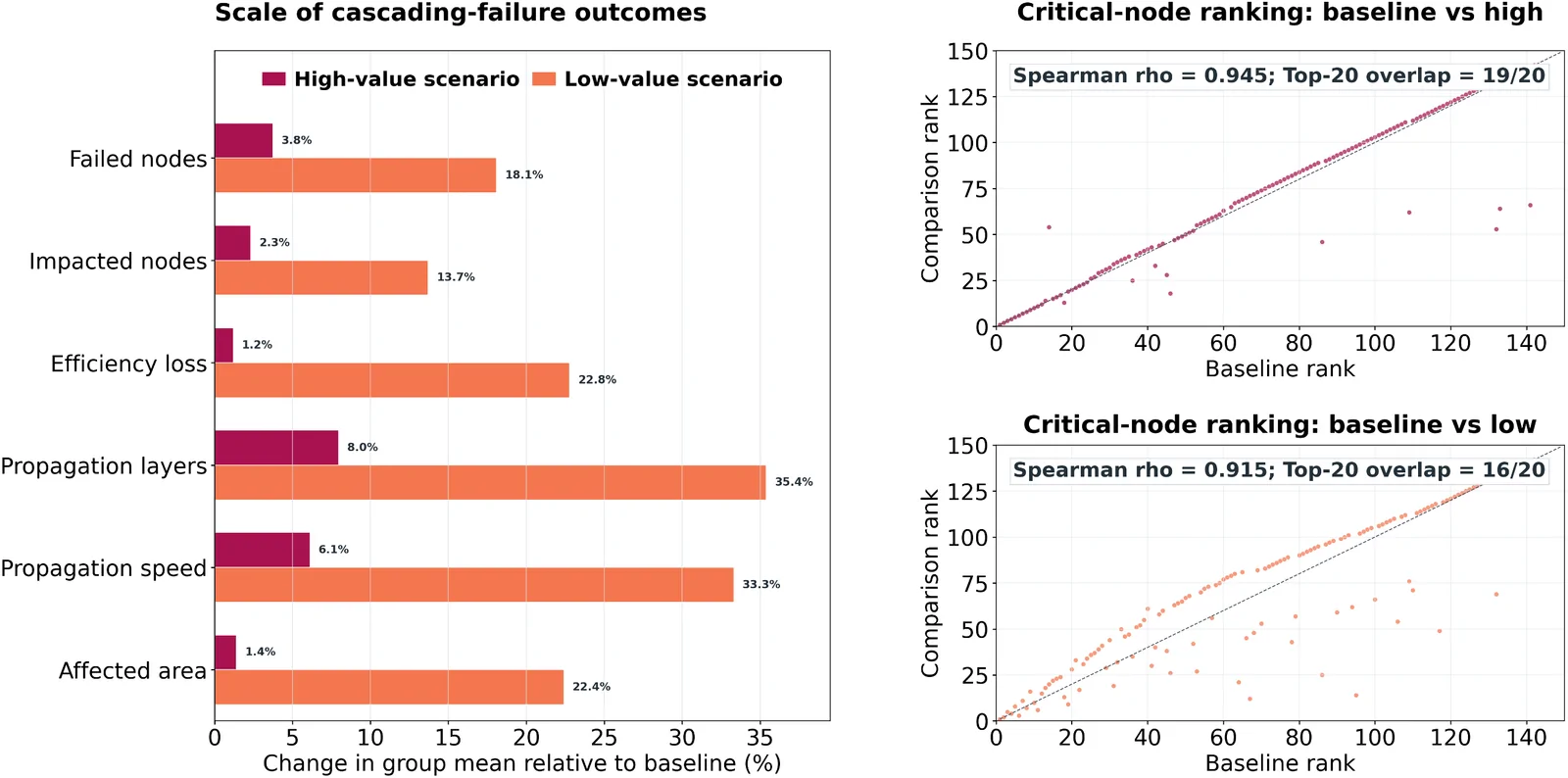
Model robustness analysis.

Despite changes in the scale of cascading failures, the ranking of reservoir nodes based on the composite criticality indicator remained highly stable. The Spearman correlation coefficients between the rankings obtained for the high-value and low-value scenarios and that of the baseline scenario were 0.945 and 0.915, respectively, while the overlap rates of their Top 20 critical reservoirs were 95% and 80%. Sanmenxia Reservoir ranked first under all three parameter scenarios, and its failure scale, spatial propagation range, and major propagation paths remained consistent. Under the three scenarios, the Top 20 nodes accounted for 62.5%, 60.7%, and 53.8% of the newly failed nodes, respectively, indicating that the head-concentration phenomenon in risk propagation persisted across different parameter settings.

### Reverse cascading failure simulation

As Zhengzhou is the core region of Henan Province where population, industry and water demand are concentrated, fluctuations in domestic water consumption, agricultural irrigation and industrial water use are more pronounced. Consequently, conducting a cascade failure simulation at the consumer end in Zhengzhou is highly representative and of practical significance. On this basis, Zhengzhou was selected for a detailed analysis of changes in the reservoir node pressure sensitivity index. The process of risk propagation is analyzed using the Liuwan Water Treatment Plant,a consumer node of relatively large design capacity within the Zhengzhou region, as the initial risk source. Furthermore, user end nodes are concentrated in specific zones within the water network. In practice, demand pressure often manifests as simultaneous fluctuations across multiple supply units within a city or a specific water supply zone. Consequently, comprehensive sensitivity indices for reservoir node pressure were calculated for each city.

#### Analysis of propagation patterns.

Fig 10 illustrates the variation in the reservoir node pressure sensitivity index as a function of the number of propagation steps following a disturbance at a single user node. A detailed analysis of the risk propagation process is presented using a demand offset of 0.25. In the Fig, the pink dotted arrows indicate the risk propagation paths, the thickness of the lines represents the probability of risk transmission, and the hollow markers indicate that the node has begun to experience pressure. It can be seen that the four reservoirs, Houhu, Laoguan Zhai, He Wang and Tanggang, immediately come under pressure, with pressure indices of 1, 0.128, 0.138 and0.036 respectively. The Houhu Reservoir reaches its pressure threshold first and, in the next step, transfers the risk of water volume exceeding the limit to other reservoirs. Following the demand pressure it receives, the pressure index for the Changzhuang Reservoir becomes 0.054, while that for the Jiangang Reservoir becomes 0.002. As the pressure resulting from exceeding the limit has been absorbed by other nodes, the pressure index for the Laoguan Zhai Reservoir node does not rise, despite its pressure connection to the Houhu Reservoir node.

**Fig 10:**
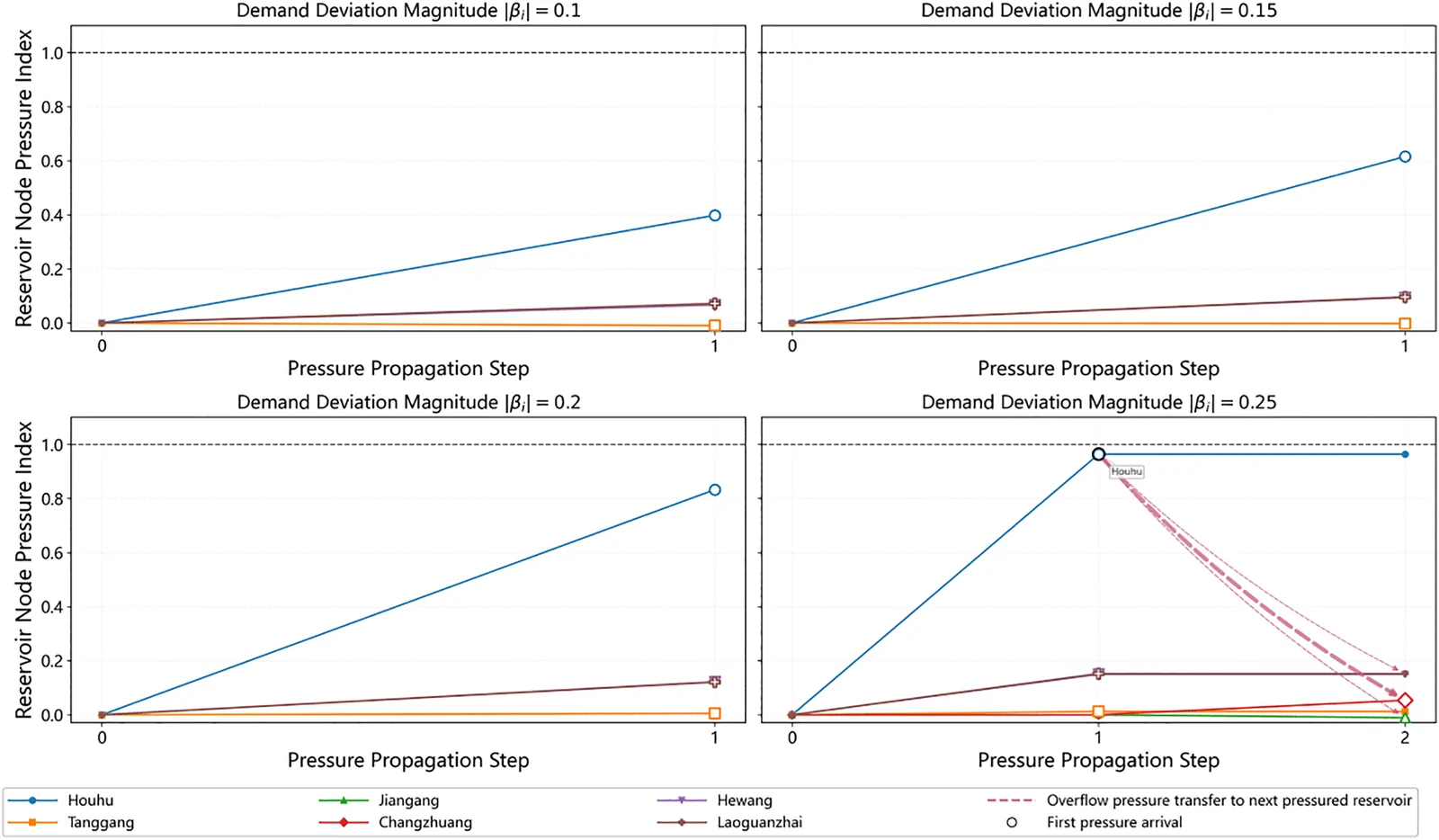
Analysis of transmission patterns.

Given that significant demand deviations do not typically occur during normal operational conditions, the demand deviation range was set to (0, 0.25], with this limit derived from Zhengzhou’s 2020 Water Resources Bulletin, to analyse the patterns of risk propagation. As the demand deviation increased from 0.1 to 0.25, the scope of water volume pressure risk propagation gradually expanded, and the pressure indices of the associated nodes all increased, in some cases even exceeding the limit. When the demand deviation was 0.1, 0.15 and 0.2, respectively, no reservoir node reached the pressure limit. When the demand deviation increased to 0.25, the Houhu Reservoir was the first to reach the pressure limit, and the excess pressure was further transmitted. After the Changzhuang Reservoir and Jiangang Reservoir absorbed the excess pressure load, their pressure indices rose. This indicates that, under a single user disturbance scenario, the propagation of water volume and pressure risks exhibits distinct hierarchical characteristics: low intensity risk disturbances primarily affect a small number of nearby reservoir nodes, while high intensity risk disturbances propagate the risk to secondary reservoir nodes connected to the nearby source reservoir nodes.

#### Analysis of reservoir node pressure sensitivity.

To analyse the pressure sensitivity of reservoir nodes, a pressure sensitivity analysis was conducted for each city to derive a comprehensive pressure sensitivity index for the reservoir nodes. Here, Zhengzhou is taken as an example, and the pressure sensitivity analysis of reservoir nodes is carried out based on two dimensions: demand deviation and disturbance intensity (Fig 11). The demand offset magnitude (${|\beta }_{i}|$) was set to the interval (0, 0.25], while the disturbance scale (k) was set to 25%, 50%, 75% and 100% of the number of user nodes in the region. The results of the sensitivity analysis for different disturbance scales indicate that both the demand offset magnitude and the disturbance scale jointly influence reservoir pressure. When the number of disturbed users is 3, the demand offset magnitude threshold is 0.15. When the number of disturbed users increases to 6, the demand offset threshold is reduced to 0.1. When the number of disturbed users reaches 12, the demand offset threshold is further reduced to 0.08. This indicates that an increase in the number of disturbed users within the region lowers the critical demand offset at which the system enters an overloaded state.

**Fig 11:**
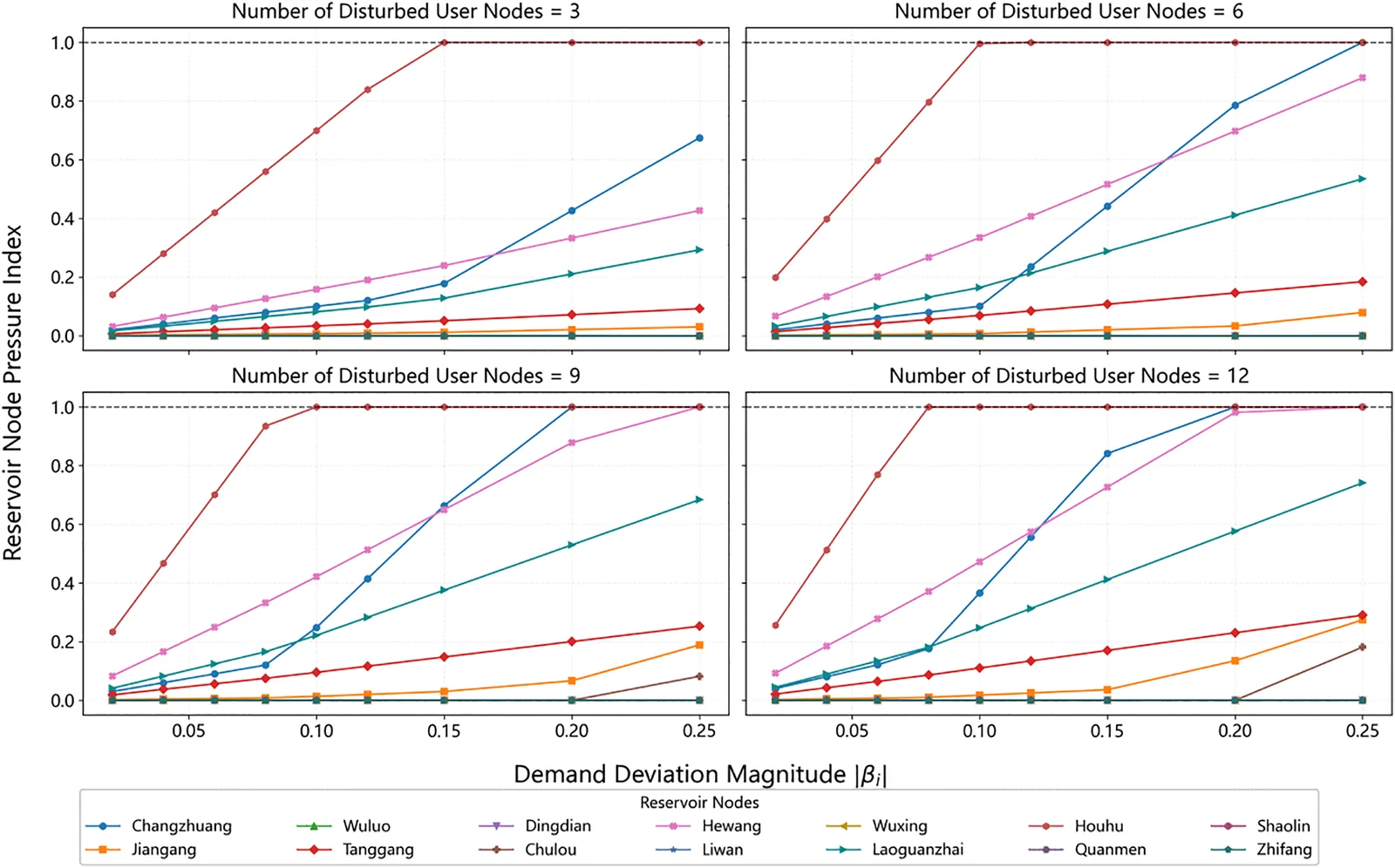
Sensitivity analysis diagram of the pressure index.

Furthermore, as the number of disturbed user nodes and the demand deviation magnitude increase, the number of reservoir nodes reaching their stress limit rises. When the demand deviation magnitude is 0.25 and the number of disturbed users increases from 3 to 12, the number of reservoirs reaching their stress limit rises from 1 to 3, while the number of reservoirs under stress increases from 4 to 7. The impact of the demand offset magnitude on the reservoir pressure index is even more pronounced. Even minor fluctuations in the demand offset magnitude can lead to a rapid rise in the reservoir pressure index.

Overall, the Houhu, Changzhuang, Hewang and Laoguan Zhai reservoirs all reached their stress limits under multiple disturbance scenarios. Based on the results of the comprehensive sensitivity index calculations, there are significant differences in the stress sensitivity of reservoir nodes within the region. Houhu Reservoir exhibits the highest comprehensive sensitivity at 4.767, while Changzhuang Reservoir, Hewang Reservoir and Laoguan Zhai Reservoir have comprehensive sensitivities of 3.945, 2.788 and 2.835 respectively. Tanggang Reservoir and Jiangang Reservoir have comprehensive sensitivities of 0.793 and 0.465 respectively. Although Wuxing Reservoir, Chulou Reservoir, Liwan Reservoir and Dingdian Reservoir reached their pressure limits under high intensity disturbances, their overall sensitivity is relatively low, indicating that they act as receiving nodes following the transmission of water volume pressure risks.

### Comparative analysis of bidirectional risk transmission

Following the bidirectional transmission simulation, two indicators were derived: the comprehensive indicator for the criticality assessment of reservoir nodes and the comprehensive indicator for the pressure sensitivity of reservoir nodes, and both indicators are used to evaluate reservoir nodes. To analyse the correlation between the two sets of results, Spearman correlation analysis was employed, revealing a weak correlation between them, with a correlation coefficient of only 0.2447.

The K-means clustering method was employed to group the data. To determine the numbers of clusters, candidate solutions with k = 2 to k = 8were evaluated using the silhouette score. Considering that the classification was intended to support the differentiated management of reservoir nodes, a minimum cluster-size constraint was also introduced to avoid management categories represented by only a small number of nodes. Specifically, each cluster was required to contain at least 10% of the 149 reservoir nodes. For the comprehensive criticality indicator, only the two-cluster solution satisfied this requirement, producing a silhouette score of 0.6997 and cluster centres of 0.0509 and 0.3447. For the comprehensive pressure sensitivity indicator, the two-, three- and four-cluster solutions satisfied the minimum cluster-size requirement, with silhouette scores of 0.7616, 0.7664 and 0.7625, respectively. Therefore, the three-cluster solution had the highest silhouette score among the admissible solutions, with cluster centres of 0.1216, 2.1481 and 5.0816. Accordingly, the comprehensive criticality indicator was divided into two groups and the comprehensive pressure sensitivity indicator into three groups. Their cross-classification generated six categories. The classification basis is detailed in Table 4.The six categories are detailed in Table 5.

**Table 4:** The result of the number of candidate classifications.

k	Criticality silhouett	minimum cluster-size	Sensitivity silhouette	minimum cluster-size
2	0.6997	26	0.7616	26
3	0.7726	13	0.7664	24
4	0.7869	6	0.7625	19
5	0.7908	1	0.7626	10
6	0.7944	1	0.7755	7
7	0.7770	1	0.7491	7
8	0.6659	1	0.7583	7

**Table 5:** Classification details table.

Partition	Number of Nodes	Proportion	Metric Range	
			G	S
High criticality – high pressure sensitivity (I)	3	2.01%	[0.2038, 0.6773]	[3.5025, 5.6950]
High criticality – medium pressure sensitivity (II)	5	3.36%	[0.2038, 0.6773]	[1.3661, 3.5025)
High criticality – low pressure sensitivity (III)	18	12.08%	[0.2038, 0.6773]	[0, 1.3661)
Low criticality – high pressure sensitivity (IV)	20	13.42%	[0, 0.1891]	[3.5025, 5.6950]
Low criticality – medium pressure sensitivity (V)	25	16.78%	[0, 0.1891]	[1.3661, 3.5025)
Low criticality – low pressure sensitivity (VI)	78	52.35%	[0,0.1891]	[0, 1.3661)

The distribution of reservoir nodes across different prefectures is shown in Fig 12. As can be seen from the Fig, reservoirs in Zone (I) with high criticality and high sensitivity are concentrated in Luoyang and Pingdingshan. Reservoirs in Zone (II) with high criticality and medium sensitivity are concentrated in Nanyang, Luoyang and shangqiu. Zone (III) high critical, low sensitivity reservoirs are mainly distributed across the south western cities. Zones (IV) and (V) low critical, medium and high sensitivity reservoirs are mainly distributed in Xinyang, Nanyang, Pingdingshan, Zhengzhou and Anyang.

**Fig 12:**
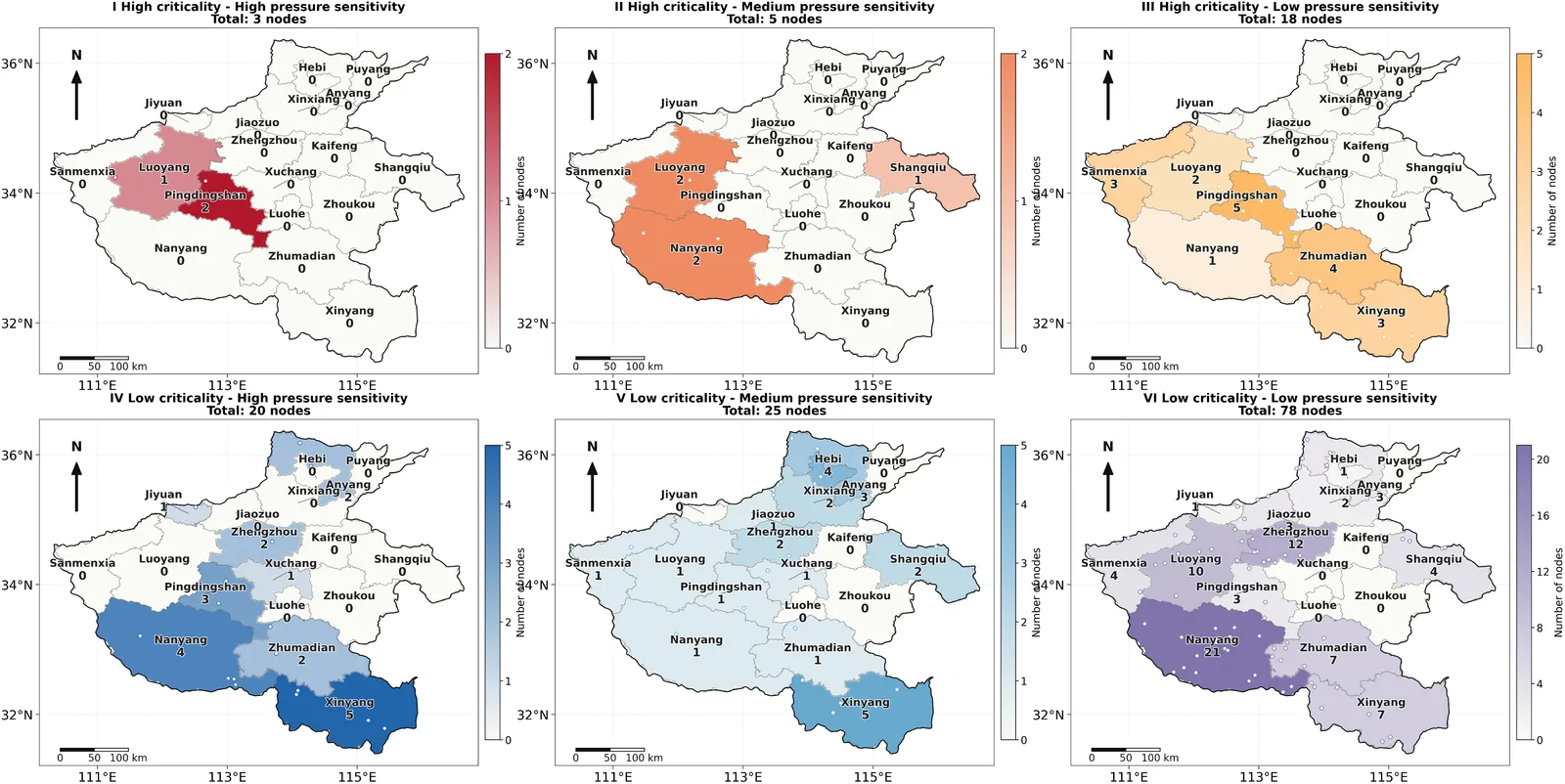
Distribution map of reservoir nodes. The provincial and prefecture-level administrative boundaries were derived from the EPS-format standard map downloaded from the National Standard Map Service of China (http://bzdt.nasg.gov.cn/indexjsp) No proprietary basemap or satellite imagery was used.

Reservoir nodes in Zones I and II exhibit high criticality combined with high or medium pressure sensitivity and therefore require priority attention. All reservoir nodes in these two zones and their corresponding indicator values are presented in Table 6. The complete classification results for all reservoir nodes are provided in S1 File in the Supporting Information.

**Table 6:** List of reservoir nodes in zones I and II.

Reservoir	Region	G	S	Category
Jianshankou Reservoir	Pingdingshan City	0.368903	5.347273	I
Guxian Reservoir	Luoyang City	0.365534	4.808703	I
Laohudong Reservoir	Pingdingshan City	0.250348	5.451868	I
Luhun Reservoir	Luoyang City	0.543873	3.302184	II
Xixiayuan Reservoir	Luoyang City	0.499667	2.733572	II
Shimen Reservoir (Nanyang)	Nanyang City	0.282512	2.661513	II
Yahekou Reservoir	Nanyang City	0.265463	2.428563	II
Zhengge Reservoir	Shangqiu City	0.218840	3.181767	II

The eight priority nodes all had G values within the high-criticality interval of 0.2038–0.6773. Because G equally integrates the normalized number of failed nodes, propagation speed, spatial propagation range, and network-efficiency loss, their high criticality reflects the combined consequences of cascading failure. The high or medium S values of nodes indicate rapid changes in reservoir pressure under variations in demand deviation and disturbance scale, which are associated with their relatively direct, low-resistance connections to user nodes.

To further demonstrate the practical applicability of the model, the reservoir nodes identified as Zones I and II were compared with publicly available water resources bulletins, engineering operation records, and documents issued by management authorities. The records used for comparison were not included in the model construction or indicator calculation. Several representative real-world cases were identified:

(1) Laohudong Reservoir was classified into Zone I, with a comprehensive criticality indicator of 0.250 and a comprehensive pressure sensitivity indicator of 5.452. According to the Pingdingshan Water Resources Bulletin, the reservoir had no stored water at the end of 2024 and was in a dry state, while reinforcement works were also being implemented. The local drought mitigation and disaster reduction emergency plan further identifies the reservoir as one of the rural drinking water sources. These actual operating conditions indicate that Laohudong Reservoir performs a water-supply function while facing management pressure associated with water storage and engineering safety, which is consistent with its identification by the model as a node with high criticality and high pressure sensitivity.
(2) Guxian Reservoir was also classified into Zone I, with a comprehensive criticality indicator of 0.366 and a comprehensive pressure sensitivity indicator of 4.809. Engineering operation records indicate that the Guxian Reservoir Water Diversion Project is responsible for providing a continuous and stable water supply to the Luoyang High-tech Water Treatment Plant and Guanlin Water Treatment Plant, with an annual water-supply target of 36.5 million m³. The management authorities have also designated Guxian Reservoir as a key target for drinking-water-source risk monitoring, emergency planning, and emergency drills. These actual water-supply responsibilities and management requirements are consistent with the high management priority identified by the model.
(3) Third, Luhun Reservoir was classified into Zone II, with a comprehensive criticality indicator of 0.544 and a comprehensive pressure sensitivity indicator of 3.302. Official records show that the Luhun Reservoir irrigation district extends across Luoyang, Pingdingshan, and Zhengzhou, covering seven counties or county-level cities, with a designed irrigation area of approximately $\text{8}.\text{949}\cdot {\text{10}}^{\text{8}}{\text{m}}^{\text{2}}$. During the spring drought in 2023, the reservoir released approximately 60 million ${\text{m}}^{\text{3}}$ of water over a period of 25 days to irrigate approximately $\text{2}.\text{267}\cdot {\text{10}}^{\text{8}}{\text{m}}^{\text{2}}$of farmland in the three cities. The implementation of drought-relief operation in response to changes in water demand is consistent with the model’s identification of the node as having medium pressure sensitivity.

## Discussion

This study focuses on the identification of risk propagation patterns, as well as the classification and differentiated management of water source nodes within complex water supply networks. A topological network model reflecting actual water supply relationships was constructed, and reservoir nodes were evaluated from two dimensions: forward risk propagation and reverse pressure feedback. Firstly, forward cascade failure simulations were conducted using reservoir nodes as the initial risk sources. The consequences of risk were characterized in terms of the number of failed nodes, propagation speed, spatial spread, and loss of network efficiency, thereby establishing a comprehensive evaluation index for the criticality of reservoir nodes. Secondly, network edges and their attributes were redefined to construct a pressure feedback network. Reverse cascade failure simulations were conducted with user nodes as the initial risk sources. The patterns of reverse transmission of demand fluctuations were revealed through changes in the pressure index of reservoir nodes as the number of propagation steps increased, and a comprehensive indicator of pressure sensitivity for reservoir nodes was derived through sensitivity analysis. Finally, by integrating the forward criticality and reverse pressure sensitivity of reservoir nodes, a two-dimensional classification is performed using the K-means clustering method, providing a basis for formulating differentiated risk management strategies for different types of reservoir nodes.

In this paper, within the framework of the forward-improved cascading failure model setting, both the structural indicators used in the traditional network structure feature analysis as the structural attributes of the nodes and the functional attributes of the nodes are taken into account. Then, Compared with degree centrality and betweenness centrality, which characterize the static topological importance of nodes, the composite criticality indicator used in this study reflects the dynamic consequences of reservoir failure in terms of failure scale, propagation speed, spatial influence, and network-efficiency loss. Furthermore, existing studies identify critical reservoir nodes based on the consequences of forward risk propagation, this study introduces reverse pressure feedback caused by demand disturbances at the user end, thereby expanding the analytical dimension of reservoir nodes from a single criterion of criticality to a dual dimension of criticality and sensitivity. This method not only identifies the impact of reservoir node failure on the water supply network but also reflects the extent to which reservoir nodes are affected by demand disturbances, thereby providing a more comprehensive characterization of their risk characteristics. The results of the Spearman correlation analysis between the two composite indicators indicate a weak correlation between the composite indicator of reservoir node criticality and that of pressure sensitivity. This is because the two indicators reflect risk propagation characteristics in different directions: the criticality composite indicator focuses on the potential consequences of cascading failures when a reservoir node acts as an initial risk source, while the pressure sensitivity composite indicator focuses on the extent to which a reservoir node is affected by demand disturbances at the user end. The weak correlation between the two further illustrates that identifying critical nodes based on a single dimension alone makes it difficult to fully characterize the risk attributes of reservoir nodes, and that it is necessary to conduct a comprehensive analysis of bidirectional propagation.

The “criticality+sensitivity” two-dimensional classification provides a practical basis for the differentiated management of reservoir nodes. Criticality reflects the cascading failure consequences caused by a reservoir node acting as an initial risk source. It can therefore be used to determine the priority of structural safety monitoring, preventive maintenance, protection of key water-conveyance routes, emergency resource allocation and infrastructure modernization. Pressure sensitivity reflects the susceptibility of a reservoir node to demand fluctuations at user nodes. It can therefore be used to determine the intensity of inflow and water-demand monitoring, pressure-index warning and operational regulation. For nodes with high pressure sensitivity, joint dispatch, coordinated supply from adjacent reservoirs or backup water sources should be activated when the pressure index approaches its threshold. Accordingly, the management intensity and priority of each zone are jointly determined by its levels of criticality and pressure sensitivity. Nodes in Zones (I), (II), and (III) all have high criticality and should be regarded as priority targets for engineering safety protection and infrastructure modernization. Specifically, priority monitoring should cover dam deformation, seepage, cracks, gates and gate-operating equipment, water-conveyance structures, and power-supply and communication facilities. Preventive inspections and equipment maintenance should also be strengthened, and emergency repair teams, equipment, and materials should be allocated in advance. In addition, the availability of alternative water-conveyance routes should be assessed, and water-network interconnection projects, backup water-conveyance routes, or emergency water sources should be developed according to actual needs to reduce the risk of cascading failures caused by the failure of critical nodes.The difference among nodes in Zones (I), (II), and (III) lies in their successively decreasing pressure sensitivity. Nodes in Zones (I) and (II) have high and medium pressure sensitivity, respectively. Therefore, in addition to the aforementioned engineering safety protection measures, changes in agricultural, domestic, industrial, and ecological water demand should be monitored by sector and region. Inflow, reservoir storage, water supply, downstream water demand, supply–demand deficits, and the pressure index should also be monitored simultaneously. When the pressure index continuously increases or approaches its threshold, joint operation of adjacent reservoirs, interregional water allocation, or backup water-source supply should be activated. If the existing water-supply capacity remains insufficient to meet demand, graded water-supply restrictions, off-peak water supply, or rotational irrigation can be further implemented. The monitoring frequency and warning-response intensity for Zone (I) nodes should be higher than those for Zone (II) nodes.Nodes in Zones (IV), (V), and (VI) all have relatively low criticality and can be managed through routine structural safety inspections and equipment maintenance. The pressure sensitivity of nodes in Zones (IV), (V), and (VI) also decreases successively. Nodes in Zones (IV) and (V) have high and medium pressure sensitivity, respectively, and should therefore adopt supply—demand monitoring, pressure warning, and operational regulation measures corresponding to those applied to Zones (I) and (II). The monitoring and regulation intensity for Zone (IV) nodes should be higher than that for Zone (V) nodes. Nodes in Zones (III) and (VI) both have low pressure sensitivity, and therefore do not require excessive resource input for water-demand monitoring.

The generality of the proposed model is primarily reflected in its modelling framework and analytical methods. The following components can be directly applied to the analysis of bidirectional risk transmission in other regional water networks without modifying the model: the analytical framework for bidirectional risk transmission, the bidirectional application of the cascading failure model, the method for determining the initial risk loads of different node types, and the setting of node risk thresholds. During model transfer, the nodes, edges, and their connections need to be reconstructed according to the local water network, and the model parameters need to be assigned using local data. For example, the storage capacities of reservoir nodes, the designed scales of user nodes, water-conveyance-route lengths, roughness coefficients, initial node risk loads need to be redetermined using local data. These adjustments constitute updates to model input data and parameters rather than modifications to the model structure. When new types of water network nodes appear in other water networks—such as when the water source node is a seawater desalination plant—it is necessary to add node attributes and define the relationships between this node and other nodes within the network.

This study generalizes the changes in water supply and water consumption caused by different water volume risks and is based on a static network topology, without considering seasonal variability in hydrological conditions, changes in management regimes or operators’ operational decisions. Future research could further refine the relationships between specific risks and differential water volumes and improve the model by incorporating dynamic networks, hydraulic simulations, seasonal hydrological conditions, changes in management regimes, operators’ operational decisions and actual dispatch data. This will enhance its ability to characterize bidirectional risk transmission under different risk and operating scenarios and thereby provide more comprehensive and in-depth suggestions for water network management.

## Conclusions

Based on the management requirements for water source nodes in complex water network, this study has established a classification and management framework for water source nodes that takes into account both the criticality of forward risk propagation and the sensitivity to reverse pressure. The conclusions of the study are as follows:

(1) The forward transmission of risks at reservoir nodes exhibits significant non-equilibrium, phased and hierarchical characteristics. A small number of reservoir nodes can trigger large scale cascading failures, and the number of ultimately failing nodes displays a pronounced ‘head heavy’ distribution, indicating that water network risks are not determined by an even distribution of nodes, but are instead concentrated and controlled by key reservoir nodes. The propagation path is not a simple single-chain diffusion, but rather first expands along the backbone connections of the water network towards structural levels, and subsequently permeates towards functional endpoints such as users, forming a transmission pattern of ‘structural diffusion functional permeation’. The propagation speed exhibits a phased change, accelerating initially and then slowing down, indicating that in the early stages, risk is primarily driven by network connectivity, while in later stages, as the load bearing status of nodes changes, risk propagation gradually attenuates.
(2) The reverse transmission of risk at user nodes exhibits distinct spatial proximity and hierarchical progression. Pressure feedback first acts upon upstream nodes closely linked to the risk source, exhibiting the characteristic of priority response by nodes near the source. It then feeds back layer by layer to upstream reservoirs along water supply relationships, forming a reverse propagation process characterized by local pressure disturbances, stepwise upstream feedback and cumulative pressure on reservoirs. Consequently, the scale of user nodes and their topological connections with reservoirs jointly determine the intensity and scope of pressure feedback.
(3) Forward water volume risk propagation and reverse pressure feedback differ markedly in terms of propagation direction, affected entities and mechanisms of action. However, both exhibit the characteristic of risk impacts concentrating on a small number of key reservoirs. The results of forward propagation reflect the capacity of a reservoir failure to impact the downstream water supply system, while the results of reverse propagation reflect the extent to which a reservoir can withstand downstream demand disturbances and pressure feedback. A comparison of the two indicates that a reservoir’s risk emitting capacity is inconsistent with its risk bearing capacity. Consequently, relying solely on a unidirectional assessment may lead to a misinterpretation of reservoir characteristics, thereby affecting the precise management of reservoir nodes.
(4) Based on the results of K-means clustering using comprehensive indicators of reservoir node criticality and sensitivity, reservoirs were classified into six categories. High criticality nodes should prioritize preventing themselves from becoming initial risk sources and mitigating the consequences of cascading failures: among these, high sensitivity nodes must balance facility safety, demand early warning, capacity redundancy and the safeguarding of backup water sources; medium sensitivity nodes should strengthen preventive maintenance of critical facilities and early warning systems for pressure related issues; low sensitivity nodes do not require specific measures regarding sensitivity, but should prioritize preventing themselves from becoming an initial risk source. As the cascading impact of low criticality nodes is relatively limited, management priorities should be adjusted progressively in line with decreasing sensitivity: high sensitivity nodes should focus on early warning of demand fluctuations, dynamic scheduling and the development of alternative supply routes; medium sensitivity nodes should prioritize periodic monitoring, initiating coordinated water supply and regional redistribution when stress levels approach thresholds; low sensitivity nodes require only routine inspections and basic maintenance. This classification more accurately identifies the characteristics of reservoir nodes and provides a basis for differentiated management of water source nodes within complex water network.

This paper constructs the topological network as a directed weighted network that takes into account the structural and functional attributes of nodes as well as the weight attributes of edges, thereby making the topological network more closely aligned with the reality of water network. It analyses the bidirectional transmission patterns of risk within water network, examines the characteristics of reservoir nodes from the dual dimensions of criticality and sensitivity, and proposes a differentiated management strategy based on classification.The limitations of this study lie in the fact that it generalizes the changes in water supply and water consumption caused by various risks using differential water volumes, which is not sufficiently detailed. Subsequent research could further refine the relationship between specific risks and changes in water volume. Furthermore, as this study has not yet incorporated dynamic hydraulic processes, future work could extend and refine the model by integrating dynamic networks, hydraulic simulations and actual dispatch data.

## Supporting information

S1 File Data supporting the construction and analysis of the Henan Province water network. The ZIP archive contains files for reservoir nodes, watercourse junction nodes, processed watercourse data, water-use nodes, reservoir-node matching, and the six-zone sensitivity classification used in the forward cascading-failure and reverse pressure-feedback analyses.
(ZIP)

S2 File Source code supporting water-network construction and bidirectional risk- transmission analysis. The ZIP archive contains Python scripts for municipal and provincial topological-network construction, the improved forward and reverse cascading-failure models, K-means clustering, and the generated municipal topology maps.
(ZIP)
